# Selenium‐Polysaccharides: Structural Characterization, Biological Activities, and Emerging Applications

**DOI:** 10.1002/fsn3.70930

**Published:** 2025-09-11

**Authors:** Shahidin, Yilong Wu, Yan Wang, Taixia Chen, Pengyan Zhu, Xuanjun Wang, Chengting Zi

**Affiliations:** ^1^ Key Laboratory of Pu‐erh Tea Science, Ministry of Education, College of Food Science and Technology Yunnan Agricultural University Kunming China; ^2^ Research Center for Agricultural Chemistry, College of Science Yunnan Agricultural University Kunming China; ^3^ College of Resources, Environment, and Chemistry Chuxiong Normal University Chuxiong China

**Keywords:** biological activities, biosynthetic pathway, emerging applications, preparation, selenium‐polysaccharides, structural characterization, structure–activity relationship

## Abstract

Selenium‐polysaccharides (SePs) are an emerging class of bioactive compounds formed by incorporating inorganic Se into polysaccharides, exhibiting enhanced biological activity compared to inorganic Se or isolated polysaccharides. The extraction of SePs from Se‐enriched sources and the selenylation of polysaccharides are crucial for improving their biological activities, leading to structural variations that contribute to diverse biological effects. Structural variations in SePs, influenced by the incorporation of Se at specific positions or the formation of seleno‐groups, play a key role in their biological effects. SePs have demonstrated antioxidant, anti‐cancer, immune‐regulating, anti‐inflammatory, and other health benefits, making them promising candidates for nutraceutical applications. However, several areas remain underexplored in existing reviews, such as the dynamics of Se and its biosynthetic pathways in plants, as well as the structure–activity relationships and emerging applications of SePs. This study aims to investigate these biosynthetic pathways, structure–activity relationships, and the potential therapeutic and industrial applications of SePs. It highlights their potential in the food and health sectors, while also emphasizing the need for further research in these areas.

## Graphical abstract

This review discusses selenium‐polysaccharides (SePs), an emerging class of bioactive compounds formed by incorporating inorganic selenium into SePs are bioactive compounds formed by incorporating inorganic Se into polysaccharides, exhibiting enhanced biological activities such as antioxidant, anti‐cancer, and immune‐regulating effects. This study explores the biosynthetic pathways, structure‐activity relationships, and the therapeutic potential of SePs, while emphasizing the need for further research into their applications in nutraceuticals and industrial sectors.

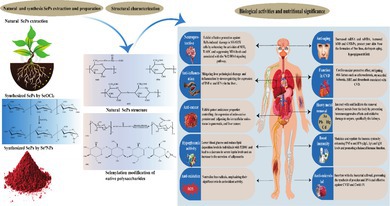

**Abbreviations**
ABTS: 2,2′‐azino‐bis(3‐ethylbenzothiazoline‐6‐sulfonic acid)
AFM: atomic force microscope
AKI: acute kidney injury
ALT: alanine transferase
APS: ATP sulfurylase (adenosine 5′‐phosphosulfate sulfurylase)
APSe: adenosine phosphoselenate
AST: aspartate transaminase
CAT: catalase
CCl4: carbon tetrachloride
CD: dichroism spectroscopy
CP: cyclophosphamide
CVB3: coxsackievirus B3
DEAE: diethylaminoethyl
DMDSe: dimethyldiselenide
DPPH: diphenyl‐1‐picryl‐hydrazyl
EMT: epithelial‐to‐mesenchymal transition
ER: endoplasmic reticulum
FT‐IR: Fourier transform infrared
Gal: galactose
GA‐SA: glacial acetic acid and selenous acid
GC: gas chromatography
GFAAS: graphite furnace atomic absorption spectroscopy
Glc: glucose
GlcA: glucuronic acid
GPxs/GSH‐Px: glutathione peroxidases
GSSG: oxidized glutathione
H_2_O_2_
: hydrogen peroxide
HO: hydroxide ion
HO^•^
: hydroxyl radical
HPGPC: high‐performance gel permeation chromatography system
HPLC: high‐performance liquid chromatography
IBD: inflammatory bowel disease
ICAM‐1: intercellular adhesion molecule‐1
ICP‐AES: inductively coupled plasma‐atomic emission spectroscopy
ICP‐OES: inductively coupled plasma optical emission spectrometry
IFN‐γ: interferon‐γ
IL: interleukin 1
IL‐1β: interleukin 1beta
LPO: lipid peroxidation
LPS: lipopolysaccharide
Man: mannose
MCF‐7: Michigan cancer foundation‐7
MDA: malondialdehyde
MeSeC: methyl‐selenocysteine
MS: mass spectrometry
Mw: molecular weight
NADPH: nicotinamide adenine dinucleotide phosphate
NA‐SA: nitric acid‐selenous acid
NA‐SS: nitric acid‐sodium selenite
NK: natural killer
NMR: nuclear magnetic resonance
O^2•–^
: superoxide anion
OAS: O‐acetylserine
PGE2: Prostaglandin E2
PHTs: phosphate transporters
ROS: reactive oxygen species
Se: selenium
Se^0^NPs: elementary Se with nanometer size
SeC: selenocysteine
SEM: scanning electron microscope
SeMet: selenium methionine
SePs: selenium‐polysaccharides
SOD: superoxide dismutase
SULTRs: sulfate transporters
T‐AOC: the total antioxidant capacity
TBAR: thiobarbituric acid reactive substances
TEM: transmission electron microscope
TNF‐α: tumor necrosis factor‐alpha
UV: UV–Vis spectrophotometer

## 1 Introduction

**FIGURE 1:**
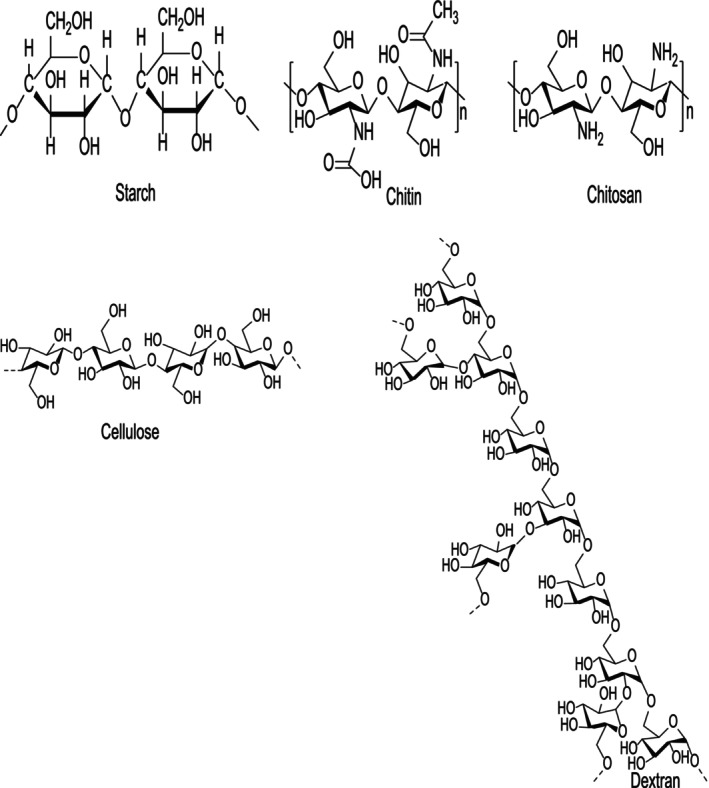
Structure of important polysaccharides including starch, chitin, chitosan, cellulose, and dextran.

Polysaccharides are large, complex carbohydrates composed of long chains of monosaccharide units linked by glycosidic bonds. They can be represented by the general formula (C_6_H_10_O_5_)_*n*_, with chain lengths ranging from 11 to several thousand units (Górska et al. 2021). They possess a more complex structure compared to other natural macromolecules, with common examples including starch, chitin, chitosan, cellulose, and dextran (Simsek et al. 2023) (Figure 1). Recent studies in medicine and functional foods have demonstrated that polysaccharides exhibit essential biological and pharmacological activities, such as antioxidant, antidiabetic, anticancer, immune‐enhancing, anticoagulant, antiviral, and hypoglycemia activities (Mohammed et al. 2021). Plant polysaccharides are widely utilized in the food, pharmaceutical, and medical industries due to their renewability, biocompatibility, and biodegradability. They are preferred over synthetic polymers for their bioactivity, homogeneity, and bioadhesive properties. These polysaccharides play a crucial role in drug delivery systems, biocomposites, and biomedical applications due to their multifunctional properties, which closely mimic those of animal and human cells (Benalaya et al. 2024). The residual side effects of synthetic drugs have shifted researchers' focus toward sustainable bio‐compounds, such as polysaccharides and their derivatives (Simsek et al. 2023). Polysaccharides perform various biological functions; however, many natural polysaccharides lack the optimal biological activity for specific therapeutic or industrial applications. To enhance the physicochemical and biological properties of natural polysaccharides, various chemical modification methods have been employed (Mukherjee et al. 2022). Selenylation, in particular, has been widely used to enhance the biological activity of polysaccharides and develop innovative sources of Se supplements (Li et al. 2022; Zhan et al. 2022).

Se is an essential trace element and dietary antioxidant, vital for growth and development in humans and animals (Zhang, Meng, et al. 2024). It was discovered in 1818 by Swedish scientist Jöns Jacob Berzelius, who named it after Selene, the Greek goddess of the moon. Se was later recognized by Schwarz and Foltz as vital for animal health (Flohé 2020), and interest in its effects on human health began to grow in the late 1960s. In nature, Se exists in two forms: inorganic Se compounds, primarily found as selenite, SeO_3_^2−^ (+4), selenate, SeO_4_^2−^ (+6), selenide, Se^2−^ (−2), and elemental Se, Se^0^ (0) (Hadrup and Ravn‐Haren 2023), while selenoprotein, selenocysteine (Hariharan and Dharmaraj 2020) and SePs (Górska et al. 2021) are organic Se compounds. Research efforts have focused on identifying human diseases that resemble Se‐responsive conditions observed in animals. Se deficiency is associated with chronic diseases, particularly those linked to inflammatory processes, such as cancer and cardiovascular disorders (Gröber and Holick 2021).

The lowest observed adverse effect level (LOAEL) for Se intake is approximately 4.3 μg/kg body weight per day (equivalent to 300 μg/day over 5 years), associated with increased mortality. The no‐observed‐adverse‐effect level (NOAEL) is 2.9 μg/kg body weight per day (approximately 200 μg/day), below which no adverse effects, including increased mortality, were observed (Hadrup and Ravn‐Haren 2023). Organic Se has been identified for over a quarter of a century as the primary source of naturally occurring Se in plant‐based foods. The bioavailability of Se is affected by its chemical form, as organic Se has higher bioavailability than inorganic forms (Zhang, Zhang, Xia, et al. 2022).

Therefore, to provide safe and efficient Se, inorganic Se can be converted into organic Se by binding with polypeptides, proteins, and polysaccharides. However, SePs show significantly higher biological activities than either Se or polysaccharides alone, as they combine the effects of both Se and polysaccharides (Table 1). They are regarded as an effective source of Se in dietary supplements due to their numerous biological activities and low toxicity (Cheng et al. 2018). SePs have gradually gained attention in research on producing functional foods and pharmaceuticals (Zhou, Long, Wang, Yu, et al. 2020). However, even in Se‐enriched areas, naturally occurring SePs are rare and often have low Se content. Due to their effectiveness and higher Se content, interest in selenylated polysaccharides has been growing. Chemically selenylated polysaccharides with varying Se content have been extensively studied. Producing Se‐enriched polysaccharides through conventional biotransformation methods is time‐consuming. As a result, synthetic approaches are being developed to generate Se‐enriched polysaccharides more efficiently and with higher Se content (Wang, Qiu, et al. 2018).

**TABLE 1:** Polysaccharides source, Se source, Se content, and biological activity of SePs.

Polysaccharide source	SePs	Se source	Se content (μg/g)	Biological activity	References
Sweet corn cob ( *Zea mays* var. *saccharate*)	SeSCP	Bio‐transformation	7.19 ± 0.067	Mitigate T2D through gut modulation	Wang, Wang, et al. (2024)
*Pleurotus ostreatus*	Se‐POP‐3	Bio‐transformation	25.9	Anti‐colon and anti‐gastric cancer	Zhang, Zhang, Liu, et al. (2022)
*Thlaspi arvense* L.	Se‐PPS1, Se‐PPS3	Bio‐transformation	13.56 ± 1.87, 15.36 ± 2.30	Antioxidant	Xiang et al. (2022)
*Boletus edulis*	Se‐BEP	Bio‐transformation	142.7	Antioxidant	Zhou, Long, Wang, Yu, et al. (2020)
Tea ( *Camellia sinensis* )	SeTPS‐1 SeTPS‐2	Bio‐transformation	23.50 13.47	Antioxidant	Gu et al. (2020)
*Pleurotus ostreatus*	Se‐POP	Bio‐transformation	3.21	Antioxidant	Zhang, Zhang, Liu, et al. (2021)
*Platycodon grandiflorum*	PGP40‐2B	Bio‐transformation	—	Anti‐cancer	
*Ginkgo biloba* L.	Se‐GBLP	Bio‐transformation	38.34	Anti‐cancer	Chen et al. (2017)
Lotus leaf (*Nymphaea* spp.)	SeLLP	Bio‐transformation	20.23	Anti‐diabetes	Zeng et al. (2017)
Corn silk ( *Zea mays* )	Se‐DCSP	Selenylated	1226.7	Anti‐inflammatory and antioxidant	Zheng et al. (2024)
*Alfalfa* ( *Medicago sativa* )	Se‐RAPS‐2	Selenylated	320	Antioxidant and anti‐tumor	Gao et al. (2020)
*Tussilago farfara* L.	STFPs	Selenylated	2338	Antioxidant	Chang and Liu (2024)
*Lycium barbarum*	SW‐SeLBP1‐1 HW‐SeLBP1‐1	Selenylated	17,490 ± 350 5680 ± 50	Antioxidant	Wei et al. (2024)
*Artemisia sphaerocephala*	SeASP	Selenylated	22,400	Suppress the growth of tumor cells	Zhu, Hu, et al. (2020)
*Alginate*	Se‐PM	Selenylated	198	Anti‐inflammation	Bi et al. (2018)
*Ulmus pumila* L.	Se‐PPUs	Selenylated	3240–13,190	Antioxidant and anti‐inflammation	Lee et al. (2017)
*Corylus mollissima*	sCPA	Selenylated	573.9	Showed a stronger ability to inhibit HeLa cell proliferation, suggesting potential as an anti‐tumor agent	Addinsall et al. (2018)
*Morchella esculenta*	Se‐Msp1	Selenylated	9560	Anti‐exercise fatigue	Addinsall et al. (2018)
Sweet potato ( *Ipomoea batatas* )	Se‐SWP	Selenylated	12,740	Antioxidant, anti‐diabetes, and antitumor	Yuan et al. (2017)
*Cordyceps sinensis*	EPS‐Se^0^NPs	Selenylated	12,740	Antioxidant, anti‐tumor, and anti‐diabetic	Yuan et al. (2017)
*Morchella sextelata*	MSP‐Se^0^NPs	Selenylated	—	Cancer chemoprevention	Shi et al. (2023)
*Paeonia lactiflora*	PLP‐Se^0^NPs	Selenylated	3910	Anti‐tumor	Wang et al. (2023)

This review aims to systematically summarize recent research on SePs, providing an inclusive understanding of their preparation methods, biosynthetic pathway, structural characterization, biological activities, and structure–activity relationship to offer valuable insights that enhance their potential applications.

## 2 Se Dynamics and Biosynthetic Pathway to Polysaccharides in Plants

Plants are an essential source of organic Se (Ren et al. 2022), primarily in the form of SePs (Table 1), which often provide various health benefits for humans (Tangjaidee et al. 2023). The formation of SePs in plants usually depends on the plant species, as well as concentration, transport, and physiological conditions such as salinity and soil pH, as well as the form and concentration of Se as it moves from the soil into the plants (Yuan et al. 2022). Additionally, the activity of membrane transporters and the plants' translocation mechanisms significantly influence Se dynamics within plants. Plants absorb inorganic Se from the soil, primarily in the forms of SeO_4_^2−^ and SeO_3_^2−^, through their roots, primarily via sulfate (SULTRs) and phosphate (PHTs) transporters (Etteieb et al. 2020). Due to its chemical similarity to S, SeO_4_^2−^ is transported in plants using the SULTRs system, while SeO_3_^2−^ is transported through PHTs (Etteieb et al. 2020). The SULTRs were first identified in SeO_4_^2−^ resistant mutants of *Arabidopsis thaliana* (Shibagaki et al. 2002). High‐affinity SULTR plays a key role in mediating SeO_4_^2−^ uptake. For instance, in Se hyperaccumulator *Stanleya pinnata* , SeO_4_^2−^ uptake is less inhibited by high sulfate pretreatment than non‐hyperaccumulators like *Stanleya elata* and *Brassica juncea* (El Mehdawi et al. 2018). Once absorbed into plant cells, SeO_4_^2−^ is transported through the root cortex and moved to the shoots via the xylem. Specific SULTRs, including SULTR2;1, SULTR2;2, SULTR3;5, and SULTR4;2, facilitate the translocation of SeO_4_^2−^ from roots to shoots (Maruyama‐Nakashita 2017). Transporters are more actively expressed in *S. pinnata* than in the non‐hyperaccumulator *Stanleya*. *elata*, highlighting the pivotal role of SULTRs in SeO_4_^2−^ transport (Wang, Cappa, et al. 2018). Similarly, the primary phosphate transporters (PHTs) responsible for SeO_3_^2−^ uptake in plants are members of the PHT1 family, which play an essential role in mediating SeO_3_^2−^ absorption due to its chemical similarity to phosphate (Cao et al. 2021). In tea plants ( *Camellia sinensis* ), genes such as PHT3;1a, PHT1;3b, PHT1;8, and the aquaporin gene NIP2;1 are upregulated in response to SeO_3_^2−^ (Ren et al. 2022).

**FIGURE 2:**
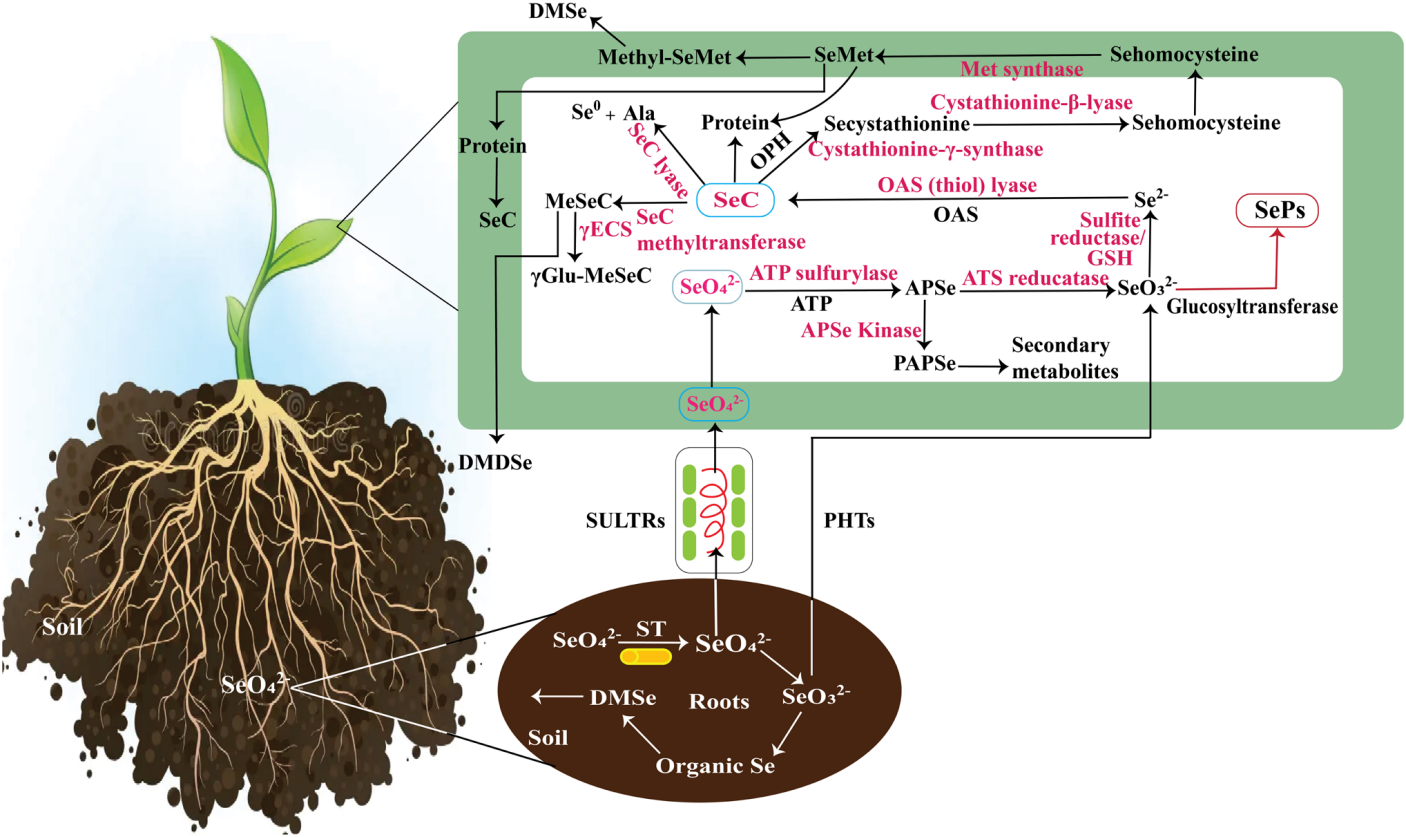
Schematic view of Se uptake and metabolism in plants: Se is absorbed by the roots through specialized transport channels, including SULTRs for SeO_4_^2−^ and PHTs for SeO_3_^2−^, and then translocated throughout the plant. Inside plant cells, Se is converted into organic compounds, such as selenocysteine (SeCys) and selenomethionine (SeMet). SePs are subsequently synthesized through enzymatic processes, likely involving glucosyltransferases (Guignardi and Schiavon 2017; Zheng et al. 2023). However, the precise cellular location of this SePs synthesis step remains unclear.

In contrast, the Se‐tolerant *Arabidopsis* mutant tps22 reduces the transcription of PHT1;1, PHT1;8, and PHT1;9 to limit Se accumulation and enhance resistance to Se toxicity (Jiang et al. 2018). Upon the absorption of SeO_4_^2−^, adenosine triphosphate sulfurylase (ATP sulfurylase, APS) catalyzes the hydrolysis of adenosine triphosphate (ATP) to form adenosine 5′‐phosphoselenate (APSe). This is followed by reduction via adenosine 5′‐phosphosulfate reductase (APR), which converts adenosine 5′‐phosphoselenate (APSe) to SeO_3_^2−^ (Hasanuzzaman et al. 2020). SeO_3_^2−^ is then converted to Se^2−^ by sulfite reductase and glutathione (GSH). The Se^2−^ is further converted into selenocysteine (SeC) in the presence of O‐acetyl serine (OAS) and OAS thiol lyase. Depending on environmental conditions, SeC may be methylated to methyl‐selenocysteine (MeSeC) by SeC methyltransferase or transformed into SeMet. SeC is incorporated into proteins as selenoproteins. In non‐hyperaccumulators, SeMet can be methylated to methyl‐selenomethionine (MeSeMet), which is then converted into non‐toxic volatile compounds such as dimethyl selenide (DMSe) or dimethyl diselenide (DMDSe), in hyperaccumulators (Guignardi and Schiavon 2017). In addition to metabolic reductions, Se is incorporated into polysaccharides through biochemical pathways likely mediated by glucosyltransferases or similar enzymes. This incorporation is believed to involve the covalent attachment of Se to carbohydrate backbones, leading to the formation of SePs (Zheng et al. 2023) (Figure 2). However, while some progress has been made in understanding the enzymatic steps involved, the detailed molecular mechanisms, regulatory factors, and metabolic pathways responsible for SePs biosynthesis in plants remain poorly understood. Unraveling these processes could provide valuable insights into plant Se metabolism and its applications in nutrition and health, emphasizing the need for further in‐depth studies.

## 3 Preparation Methods of SePs

The concentration of SePs in plants is typically low, as only a few natural SePs have been identified. Consequently, most research has focused on synthesizing SePs through chemical methods or bioaccumulation. Synthetic SePs generally have higher Se content than their natural forms, which is believed to enhance their biological activity (Gu et al. 2020). Therefore, to achieve higher concentrations of SePs, two primary methods are used: the first method involves extracting natural SePs from Se‐enriched sources, and the second method involves selenylating polysaccharides by integrating Se into native polysaccharides (Zhan et al. 2022).

### 3.1 Extraction and Purification of Natural SePs From Plants

**FIGURE 3:**
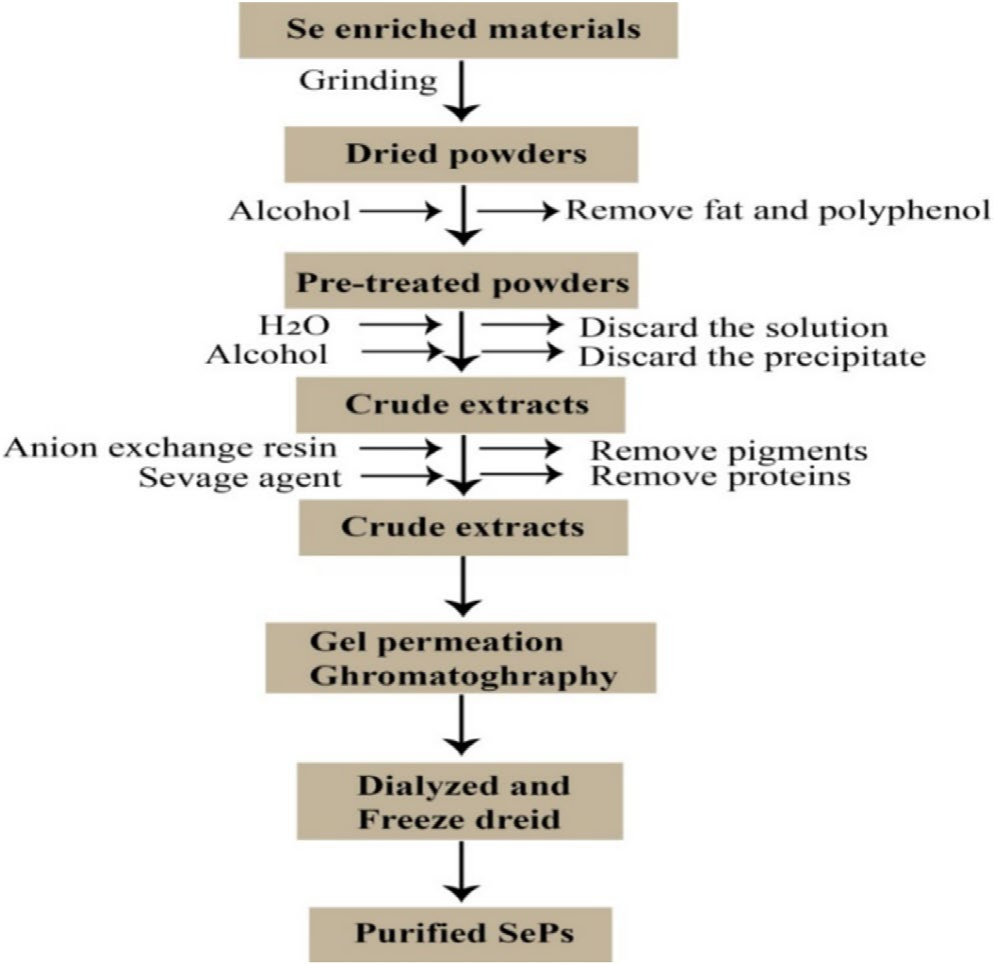
Extraction and purification of SePs from Se‐enriched materials involve grinding and alcohol extraction to remove fats and polyphenols. Crude extracts are then purified to remove pigments and proteins, followed by gel permeation chromatography, dialysis, and freeze‐drying to yield purified SePs.

SePs are extracted and purified using similar methods to those used for traditional polysaccharides. Se‐enriched materials undergo pretreatment with ethanol and petroleum ether to remove fats and pigments before SePs are extracted via water decoction and ethanol precipitation. After centrifugation and concentration, the protein is separated from the raw material solution using Savage reagents (chloroform/n‐butanol, 4:1) based on its ion exchange and hydrophilic properties. To obtain crude SePs, the solution is precipitated with ethanol, dialyzed, and then freeze‐dried. This process is naturally optimized using response surface methodology, with the assistance of techniques such as ultrasound and microwave, to achieve a high yield of the end product. The crude SePs are further purified using ion exchange, gel permeation, and size exclusion chromatography, as well as DEAE‐cellulose, Sephacryl, Sepharose, Superdex, and Sephadex (Figure 3). Generally, the purification procedure uses distilled water and a gradient NaCl solution as the eluents. The SePs fractions are analyzed using the anthrone‐sulfuric acid or phenol‐sulfuric acid method. Finally, to obtain purified natural SePs, the collected peak eluent is subjected to dialysis and freeze‐drying (Li, Shen, et al. 2019).

### 3.2 Selenylation of Natural Polysaccharides

**FIGURE 4:**
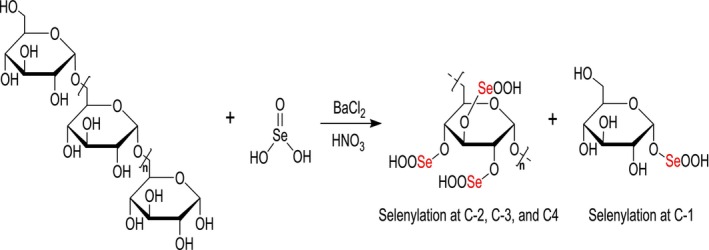
Structure of SePs produced through the HNO_3_–Na_2_SeO_3_ method, characterized by 1→6 linkages in the polysaccharide chain. Selenylation introduces ‐HSeO_3_ groups, which can bond at the C‐2, C‐3, and C‐4 positions, as well as at the C‐1 position on the reducing end of the sugar.

As mentioned earlier, naturally occurring SePs are scarce and typically have low Se content. Selenylation modification of polysaccharides is a commonly used method for producing chemically modified SePs. The native polysaccharides are mainly selenylated with selenide reagents. The most common selenylating modification methods include nitric acid‐selenous acid (NA‐SA), nitric acid‐sodium selenite (NA‐SS), glacial acetic acid‐sodium selenite (GA‐SS), glacial acetic acid and selenous acid (GA‐SA), and Selenium oxychloride (SeOCl_2_) methods to produce SePs (Duan et al. 2021). Each technique is capable of generating synthetic SePs. The NA‐SS technique is most frequently used because of its high selenylation value. This can be shown as follows: polysaccharides are added briefly to nitric acid (HNO_3_) solution while being stirred. After adding Na_2_SeO_3_ and BaCl_2_, the reaction is allowed to proceed for several hours. Subsequently, when the reaction is completed, a solution of NaOH is added to neutralize the mixture, followed by Na_2_SO_4_ to remove Ba^2+^ ions. To produce synthetic SePs, the supernatant collected after centrifugation is dialyzed and then freeze‐dried (Yang et al. 2021) (Figure 4). Numerous studies have reported the synthesis of SePs using HNO_3_–Na_2_SeO_3_ selenylation method, employing polysaccharides from various sources. SePs synthesized from sweet corncob polysaccharides were confirmed by X‐ray, which identified the incorporated Se species as the SeO_3_^2−^ group (‐OSe[O]‐OH) (Wang et al. 2022).

**FIGURE 5:**
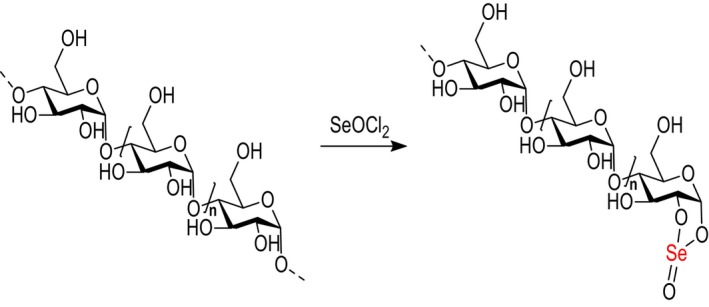
The polysaccharide is selenylated with SeOCl_2_, replacing the OH group on the Glc units with Se=O. This reaction incorporates Se into the polysaccharide, transforming it into a selenylated form (SePs).

The use of SeOCl_2_, another effective selenylating reagent, has made the process of producing SePs more efficient. Using polysaccharides derived from *Artemisia sphaerocephala*, a Se content of 22,400 μg/g can be synthesized via the SeOCl_2_ method, significantly higher than 1703 μg/g achieved with the HNO_3_/H_2_SeO_3_ selenylation method (Zhu, Hu, et al. 2020). Consequently, the SeOCl_2_ system effectively enhances the Se content in SePs (Figure 5).

The second method of selenylation modification of polysaccharides involves using Se nanoparticles (elemental Se in nanometer size, Se^0^NPs), which have been integrated into polysaccharides. Se^0^NPs are vibrant red particles produced through either biotic or abiotic reduction of Se, known for their high biological activity and low toxicity (El‐Badri et al. 2022). The synthesis of Se^0^NPs involves Na_2_SeO_3_ as the Se precursor and ascorbic acid (Vc) as the reducing agent. The Na_2_SeO_3_ solution is generally prepared at a concentration of around 100 mM, while the Vc is made fresh at 200 mM to promote effective reduction. The molar ratio of Na_2_SeO_3_ to Vc commonly ranges from 1:1 to 1:5, enhancing both reduction efficiency and nanoparticle stability. The addition of reagents to a polysaccharide suspension can be achieved through two primary methods. In the first step, Na_2_SeO_3_ is added to the polysaccharide suspension before Vc, allowing for better control over particle formation and resulting in a narrow size distribution (Cui et al. 2019). In the second method, Vc is mixed with the polysaccharide before adding Na_2_SeO_3_, which can yield distinct structural properties in the resulting nanoparticles (Hu et al. 2020). For uniform nanoparticle formation, both Na_2_SeO_3_ and Vc should be added dropwise under magnetic stirring in a dark environment to prevent oxidation and aggregation. The synthesis typically occurs between 25°C and 37°C, although higher temperatures (up to 150°C) can lead to more extensive and variable particle sizes (Bi et al. 2018). Key parameters influencing particle size and homogeneity include the Se‐to‐polysaccharide ratio and concentration. Effective polysaccharide concentrations range from 1 to 2.5 mg/mL, with higher polysaccharide proportions typically reducing nanoparticle size and enhancing uniformity. After synthesis, the Se^0^NPs generally are left to stabilize for about 24 h, followed by purification through centrifugation and washing with water and ethanol to remove residual reagents. Ultrasound can be applied during synthesis to avoid precipitation and to achieve a more uniform Se^0^NPs structure. This approach produces Se^0^NPs with diameters typically ranging from 15 to 70 nm. The application of ultrasound helps control particle size, resulting in nanoparticles with smaller diameters and a higher specific surface area. Consequently, ultrasound‐treated Se^0^NPs exhibit enhanced radical‐scavenging capabilities compared to untreated Se^0^NPs, which is attributed to their reduced size and increased surface area (Bi et al. 2018).

## 4 Structural Characteristics of SePs

### 4.1 Analytical Methods for Structural Characterization of SePs

The structural analysis of SePs involves both chemical and instrumental techniques. Chemical methods such as the Bradford assay and the phenol‐sulfuric acid method are often used to quantify proteins and carbohydrates, respectively. Instrumental methods for determining molecular weight include high‐performance gel permeation chromatography (HPGPC), Vapor pressure osmometry, and Membrane osmometry, which are commonly used. Mass spectrometry (MS), gas chromatography (GC), and high‐performance liquid chromatography (HPLC) are used to analyze the monosaccharide composition of SePs. The secondary structural arrangement of SePs is characterized using circular dichroism spectroscopy (CD). To identify the configurations of glycosidic bonds, chemical and substituent groups, surface analysis, and elemental composition, various techniques are employed, including ultraviolet (UV) spectrophotometry, X‐ray photoelectron spectroscopy (XPS), nuclear magnetic resonance (NMR), and Fourier transform infrared (FT‐IR) spectroscopy. Micromorphology of SePs is analyzed using an atomic force microscope (AFM), transmission electron microscope (TEM), and scanning electron microscope (SEM). Quantification of Se in SePs is achieved using Atomic fluorescence spectrophotometry (AFS), hydride generation atomic absorption spectrometry (HG‐AAS), hydride generation‐atomic fluorescence spectrometry (HG‐AFS), inductively coupled plasma optical emission spectrometry (ICP‐OES), inductively coupled plasma mass spectrometry (ICP‐MS), and graphite furnace atomic absorption spectroscopy (GFAAS) (Zhou, Long, Wang, Yu, et al. 2020).

### 4.2 Structural Classification and Characterization of SePs

The structural complexity of SePs arises from their hierarchical organization, compositional diversity, and unique Se integration. The primary structure of SePs is defined by monosaccharide sequences and the specific sites where Se is incorporated into the polysaccharide backbone. The secondary and tertiary structures describe the macromolecular folding of polysaccharides. In contrast, the quaternary structure involves the aggregation of polysaccharide chains, observed as spherical clusters or loose, flaky morphologies (Niaz et al. 2020).

In SePs, Se is covalently bonded to the polysaccharide chains. The structural diversity of SePs is due to variations in the insertion positions of Se within the polysaccharide backbone. These structures typically contain two types of glycosidic bonds: α‐glycosidic bonds and β‐glycosidic bonds. The composition and proportion of monosaccharides in SePs vary, including glucose (Glc), galactose (Gal), xylose (Xyl), rhamnose (Rha), mannose (Man), arabinose (Ara), galacturonic acid (GalA), etc. SePs possess a Se atom covalently linked to the aldehyde or hydroxyl (OH) groups of the polysaccharides, with weak interactions through van der Waals forces, hydrogen bonds, and salt bonds. FT‐IR, GC, GC–MS, and NMR analyses revealed the presence of →3)‐α‐D‐Glc‐(1→, →4)‐α‐D‐Glc‐(1→, →6)‐α‐D‐Man‐(1→, →6)‐β‐D‐Gal‐(1→, and →4)‐α‐L‐Rha‐(1→, and other glycosidic linkages in SeCPS‐II, with primary →4)‐α‐L‐Rha‐(1→ bonds suggesting a strong polysaccharide structure. Specific linkages, along with their substantial molecular weight of 4.12 × 10^6^ Da and Se content of 17.89 μg/g, show a complex structural composition that may contribute to SeCPS‐II's unique functional properties (Sun et al. 2018). Se‐POP‐3, derived from *Pleurotus ostreatus*, was structurally characterized, revealing a primary composition of Gal, Man, and Glc in a molar ratio of 2.4:1.7:49.6, with Se content of 25.9 μg/g and a molecular weight of approximately 1.61 × 10^4^ Da. Spectral analysis shows that Se‐POP‐3 is a pyranose polysaccharide linked by α‐glycosidic bonds in the main chain, with Se present in C‐O‐Se and Se=O forms (Zhang et al. 2020).

Se‐POP‐21, another Se‐enriched polysaccharide, was similarly characterized and contained Se in C‐O‐Se and Se=O forms within a non‐triple helix pyranopolysaccharide structure. Morphological analysis of Se‐POP‐21 revealed a spherical shape, with particle sizes ranging from 100 to 200 nm and larger clusters of 500–600 nm (Zhang, Zhang, Liu, et al. 2021). Advanced structural characterization using GC–MS and 2D NMR revealed that Se‐POP‐3 consists of →[3)‐β‐D‐Glcp‐(1]_2_→6)‐β‐D‐Glcp‐(1→3,6)‐β‐D‐Glcp‐(1→3)‐β‐D‐Glcp‐(1→, linkages. Its branching structure, containing α‐D‐Glcp‐(1→[4)‐α‐D‐Glcp‐(1]_4_→, is linked to the main chain via the O‐3 position of the →3,6)‐β‐D‐Glcp‐(1→ glycosidic bond, showing a complex and highly branched structure (Zhang, Zhang, Liu, et al. 2022).

**FIGURE 6:**
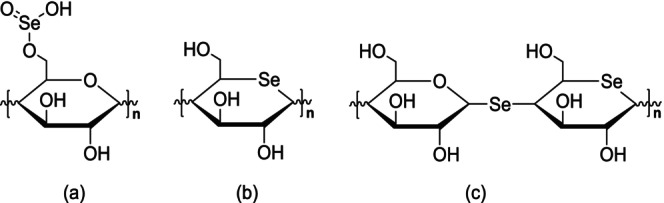
Suggested binding sites for Se in SePs. (a) Bond formation of Se=O occurs at the C‐6 position. (b) Se replaces O_2_ in the acetal ring of β‐glucose. (c) Se integration into the glycosidic linkage.

Comparative studies of natural (NSe‐TPS2) and artificial (ASe‐TPS2) Se‐enriched tea polysaccharides were conducted. Despite differences in their sources, both polysaccharides exhibited similar structural characteristics, with molecular weights of 2.44 × 10^3^ Da and 6.73 × 10^3^ Da, respectively. The chemical structure of ASe‐TPS2 primarily consists of →4)‐α‐D‐Gal*p*A‐(1→, →2)‐α‐L‐Rha*p*‐(1→, →4)‐Glc*p*‐(1→, →3)‐β‐D‐Glc*p*‐(1→ linkages, suggesting a strong backbone capable of supporting biological activity. In contrast, NSe‐TPS2 primarily contains →4)‐α‐D‐Gal*p*A‐(1→, and →4)‐β‐D‐Glc*p*‐(1→ units, with branches linked by →3)‐α‐D‐Gal*p*‐(1→, →2)‐β‐L‐Rha*p*‐(1→, and →2)‐β‐L‐Ara*p*‐(1→ linkages, indicating a potentially higher degree of branching and structural diversity (Zhu et al. 2019). Similarly, two new Se‐enriched tea polysaccharide fractions, SeTPS‐1 and SeTPS‐2, were isolated and structurally characterized with molecular weights of 17 × 10^3^ and 13 × 10^3^ Da, and Se contents of 23.50 and 13.47 μg/g, respectively. Spectral analyses revealed absorption bands characteristic of Se esters, suggesting that Se is covalently bound in the polysaccharide matrix. SeTPS‐1 consists primarily of Gal and Glc in a molar ratio of 2.3:80.1, while SeTPS‐2 comprises Glc, Gal, Ara, and GalA in a molar ratio of 48.83:3.21:2.04:1.30. Both show random coil conformations, indicative of a flexible structural form that could influence their biological interactions (Gu et al. 2020). Further studies on *Thlaspi arvense* L. separated and characterized two distinct fractions, Se‐PPS1 and Se‐PPS3, with molecular weights of 4.2 × 10^4^ and 4.5 × 10^4^ Da, respectively. Se‐PPS1 comprises Gal, Xyl, and Glc, while Se‐PPS3 contains a more complex array of monosaccharides, including GalA, GlcA, Xyl, Glc, Gal, Ara, and Rha. FT‐IR spectroscopy confirmed absorption spectra characteristic of Se esters, while GC–MS and NMR analyses revealed →6)‐β‐D‐Gal*p*‐(1→ and α‐D‐Glc*p*‐(1→ linkages in both fractions, indicating the presence of both hexose and pentose sugars in specific glycoside bonds that might affect their solubility and bioavailability (Xiang et al. 2022). Two novel homogeneous Se‐RLFP‐I and Se‐RLFP‐II were extracted from *Rhamnus laevigata*, with average molecular weights of 2.4 × 10^4^ and 1.6 × 10^4^ Da. The primary structural analysis identified both Se‐RLFPs as α‐pyranoses composed of Glc, Rha, and Xyl, suggesting a ring structure that may enhance their stability and binding potential in biological systems (Liu et al. 2022). The structural characterization of this SePs, including their molecular weights, branching patterns, and Se binding forms, for example, Se esters, highlights potential sites for Se stabilization within the polysaccharide matrix. Structural insights provide a foundational understanding of how Se interacts with the polysaccharide backbone, suggesting that specific glycosidic linkages, molecular conformations, and Se‐binding sites are likely to influence the biological and therapeutic effects of Se‐enriched polysaccharides (Figure 6).

In FT‐IR spectra of various selenylated polysaccharides, new absorption peaks were identified at 600–700 and 850–900 cm^−1^, attributed to C‐O‐Se stretching vibrations and Se=O asymmetric stretching, respectively. Under certain conditions, additional peaks were observed at 600–700 cm^−1^ (O‐Se‐C) and 1010–1040 cm^−1^ (O‐Se‐O), showing unique Se incorporation into the polysaccharide structure (Liu et al. 2018). Structural analyses confirmed that Se was successfully incorporated into the polymer chain of EJP90‐1 from *Eriobotrya japonica* leaves through Se‐O bonds, formed via Na_2_SeO_3_–HNO_3_ modification process, as verified by XPS and EDS techniques (Zhang et al. 2021). SEM imaging of SePs derived from *Medicago sativa* roots revealed a loose, flaky structure in selenized RAPS‐2, contrasting with the tight, striped appearance of native RAPS‐2. This modification involved two polysaccharides, RAPS‐1 and RAPS‐2, with molecular weights of 1.0 × 10^4^ and 1.58 × 10^4^ Da, respectively. RAPS‐1 and RAPS‐2 varies in glycosidic linkages, as RAPS‐1 includes 1→2, 1→4, 1→), and 1→6, while RAPS‐2 lacks the 1→4 linkage. Selenylation reduced the molecular weight of Se‐RAPS‐2 to 1.1 × 10^4^ Da, further influencing the polysaccharide's physical structure (Gao et al. 2020). In *Sagittaria sagittifolia* L., Se‐PSSP maintains the pyranoid structure linked by α‐glycosidic bonds. Both PSSP and Se‐PSSP exhibited amorphous morphologies and lacked a triple‐helix structure. However, selenization decreased the molecular weight from 4.712 × 10^4^ Da in PSSP to 1.682 × 10^4^ Da in Se‐PSSP, with modifications in monosaccharide composition from Xyl, Man, and Glc (14.86%, 14.35%, and 55.82%) to Xyl, Gal, and Glc (18.76%, 18.14%, and 26.49%) (Feng et al. 2022). Further structural investigations into two artificial Se‐enriched tea polysaccharides (Se‐TPS) highlighted structural differences. In ASe‐TPS1, Se substituted OH groups at C‐1 and C‐6, forming Se‐H bonds, while in CSe‐TPS1, Se replaced the OH group specifically at C‐6, forming a selenyl ester (Zhu, Yu, et al. 2020). Selenized Chinese *Angelica* polysaccharides (sCAP) exhibited molecular weights ranging from 9.0 × 10^2^ and 9.7 × 10^4^ Da, primarily consisting of Ara, Gal, and GalA. sCAP contained both α and β pyranose configurations, with Se‐O‐C vibrational peaks and GalA‐related signals, indicating structural stability even after selenylation (Qiao et al. 2024). Interestingly, for Se‐enriched *Spirulina* polysaccharides (SeCSPS), surface morphology changes were observed at Se‐binding sites. However, the primary structure remained largely consistent with the native polysaccharide SPS‐3 (Qian et al. 2024). For selenized polysaccharides from *Lonicera caerulea* L. (PSLP‐1 and PSLP‐2), a selenization process involving HNO_3_ and Na_2_SeO_3_ led to Se contents of 228 ± 24 and 353 ± 36 μg/g, respectively. Although molecular weights were slightly reduced post‐selenylation from 5.9 × 10^7^ Da in PLP to 5.6 × 10^7^ Da in PSLP‐1 and 5.1 × 10^7^ Da in PSLP‐2), the core chain structure and glycosidic linkages remained intact, indicating that selenylation selectively modified only certain regions without altering the fundamental polysaccharide backbone (Shao et al. 2023). SeOCl_2_ was a highly reactive selenide reagent synthesizing selenized *A. sphaerocephala* polysaccharides (SeASP). FT‐IR, Raman, NMR, and XPS analyses revealed that the seleno‐group was specifically substituted at the C‐6 position in the form of SeO_3_^2−^. Furthermore, SEC‐MALLS analysis showed that the SeOCl_2_ system effectively prevented degradation of the polysaccharide chain, maintaining its structural integrity.

**FIGURE 7:**
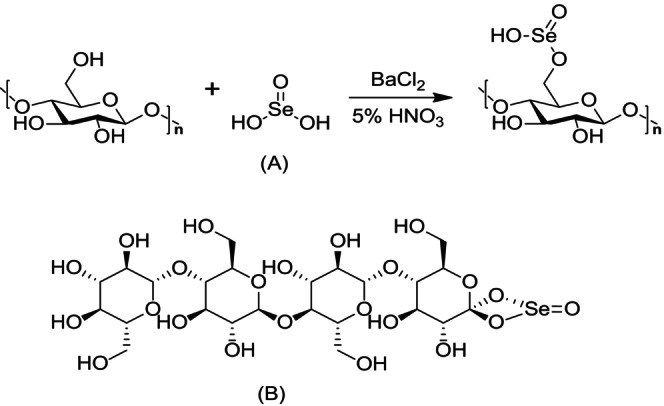
The chemical reaction process of selenylation modification of native polysaccharides and the structure of SePs derived from *Astragalus* are shown in (A) and (B), respectively.

The selenized derivative of *Sargassum pallidum* polysaccharides (Se‐SPP), with a Se content of 2419 μg/g, was synthesized and characterized. Physicochemical analyses revealed that selenylation induced changes in the chemical composition, altered the monosaccharide profile, increased molecular weight, and modified the surface morphology of the native polysaccharides. The FT‐IR spectroscopy further identified a new absorption peak at 675 cm^−1^ in Se‐SPP, likely due to the incorporation of a selenyl group (Xiao et al. 2019). These modifications highlight the structural effect of selenylation. In selenylated polysaccharides, the primary interaction with Na_2_SeO_3_ occurs at the hemiacetal hydroxyl group, where Se is generally introduced as a Se ester (Figure 7A,B).

## 5 Biological Activities of SePs

Due to their unique composition and properties, SePs exhibit diverse biological activities essential for maintaining human health. Se, primarily incorporated into SePs through diet, plays a vital role in mitigating human‐related diseases. The biological activity of SePs depends on various structural factors, including molecular mass, monosaccharide composition, and the degree of substitution. Specific structural elements largely determine how SePs carry out the biological activity attributed to Se.

### 5.1 Antioxidant Activity

Most studies on the biological activity of SePs have concentrated on their potent antioxidant properties, as shown in Table 1, which have gained significant attention due to the role of oxidative damage in cellular degeneration and disease. A primary antioxidant mechanism of SePs is their capacity to scavenge free radicals, highly reactive molecules generated within organisms that can trigger various diseases and accelerate aging. Among oxygen radicals, the hydroxyl radical (HO^•^) is primarily reactive and damaging, as it quickly penetrates cell membranes and interacts with essential macromolecules, including carbohydrates, proteins, lipids, and nucleic acids, leading to significant structural and functional damage (Lian et al. 2018). Studies show that the antioxidant activity of SePs is superior to that of Na_2_SeO_3_, selenoproteins, and unmodified polysaccharides at equivalent concentrations. This enhanced biological activity is closely linked to structural modifications, particularly the incorporation of Se into polysaccharide molecules, which optimizes their composition and function. The substantial antioxidant effects of SePs are attributed to their Se content, which distinguishes them from other Se compounds and native polysaccharides. Research evaluating the biological activity of SePs highlights their strong antioxidant capacity across various sources. For instance, SePs such as Se‐PPS1 and Se‐PPS3 influence the redox properties of Se, enhancing its antioxidant activity by scavenging free radicals and reducing oxidative stress. This antioxidant effect is further facilitated by the specific glycosidic linkages and sugar compositions, which enable the polysaccharides to interact effectively with ROS (Xiang et al. 2022). SePs derived from *P. ostreatus* (Se‐POP) demonstrated superior abilities to scavenge free radicals like ABTS, DPPH, HO^•^, and O_2_^•−^ compared to native polysaccharides (Ma et al. 2018). This enhanced scavenging activity can be partially attributed to the reduction in uronic acid content, which appears to increase the electron‐donating capacity of this modified polysaccharide, thereby amplifying its ability to neutralize oxidative species that can damage the biological macromolecules (Liu and Huang 2019). Degraded corn silk polysaccharides (DCSP) were selenized using HNO_3_–Na_2_SeO_3_ method to produce Se‐DCSP, and the resulting compounds were compared for antioxidant activity. The selenization process modified the structure of DCSP, resulting in a 2‐ to 3‐fold increase in free radical scavenging ability and a 1.5‐fold reduction in intracellular reactive oxygen species content. Additionally, structural changes decreased mitochondrial membrane potential by approximately 2.5 times, indicating enhanced cellular resilience to oxidative stress (Zheng et al. 2024).

Se, as an essential cofactor for enzymes such as SOD and glutathione peroxidase (GSH‐Px), enhances the antioxidant response by catalyzing the conversion of reactive oxygen intermediates into less harmful molecules, thereby providing a biochemical basis for the in vivo effects observed. The antioxidant role is further supported by studies on juvenile black sea bream, where SePs administered with a basal diet significantly increased the activities of catalase (CAT), GSH‐Px, and SOD in both liver and serum, leading to a reduction in ROS levels and a subsequent increase in oxidative stress resistance (Wang et al. 2019). In addition to enhancing endogenous antioxidant defense, certain SePs display direct radical scavenging abilities that depend on their specific chemical structure. For example, CEP‐Se^0^NPs involve the synergistic effect of Se and *Cyperus esculentus* polysaccharides (CEP). Se^0^NPs exhibit antioxidant activity by scavenging free radicals, while the polysaccharides in CEP further enhance this effect through their natural antioxidant properties. The physical binding of CEP to the Se^0^NPs enhances their stability, enabling sustained antioxidant action, particularly in environments such as simulated digestion (Zhai et al. 2024). Other Se‐modified polysaccharides, such as Se‐enriched *P. ostreatus* polysaccharide (Se‐POP‐21) and Se‐enriched *Hohenbuehelia serotina* polysaccharide (Se‐HSP), have likewise demonstrated strong DPPH and hydroxyl radical scavenging abilities in vitro assays (Zhang, Zhang, Liu, et al. 2021). Exceptionally, Se‐HSP maintained or enhanced its antioxidant activity without altering the fundamental properties of unmodified HSP, suggesting that Se enrichment can increase antioxidant potential without compromising structural stability. Se‐modified *T. arvense* L. polysaccharides Se‐PPS1 and Se‐PPS3 and *Morchella sextelata* SeMSP‐4 also display significant in vitro antioxidant capacities, with SeMSP‐4, in particular, exhibiting more practical radical‐scavenging abilities in vitro than native MSP‐4, demonstrating their potential as natural antioxidants (Deng et al. 2024; Xiang et al. 2022). The antioxidant mechanism of Tribonema polysaccharide (TP)‐Se^0^NPs follows a similar pathway, with TP enhancing Se^0^NPs' ability to scavenge radicals like DPPH, superoxide anions, and hydroxyl radicals (Yang et al. 2025).

### 5.2 Anti‐Cancer Activity

Since the late 1960s, observational studies have suggested that people with higher levels of Se in their diets or body tissues may have a lower risk of cancer. The increasing interest in Se supplements has led to their exploration as potential cancer preventatives (Balboni et al. 2022). Among these, SePs have gained attention for their promising anticancer properties. For instance, SePs from *Grifola frondosa* (Se‐LMW‐GFP) demonstrated more substantial anticancer effects than their native polysaccharide, effectively inhibiting the proliferation of BGC‐823 and MFC cancer cells. The mechanisms appear to involve the Fas/FasL‐mediated death receptor pathway and the intrinsic mitochondrial pathway, triggering the apoptosis of cancer cells together (Huo et al. 2024). Polysaccharide PVP3‐1 was modified with Se^0^NPs to produce PVP3‐1‐Se^0^NPs. Anti‐pancreatic cancer cell assays demonstrated that PVP3‐1‐Se^0^NPs effectively inhibited the proliferation and migration of pancreatic cancer cells in vitro, inducing apoptosis and autophagy in cancer cells by inhibiting the mTOR signaling pathway. By blocking mTOR signaling, PVP3‐1‐Se^0^NPs disrupt cellular survival and growth pathways, thereby promoting cancer cell death and reducing cell viability (Zhang, Wang, et al. 2024). Se‐POP‐3 has demonstrated in vitro anticancer activity by inducing apoptosis and inhibiting the progression of cancer cells. This effect may arise from Se‐POP‐3 ability to alter the Bax/Bcl‐2 protein ratio and inhibit epithelial‐to‐mesenchymal transition (EMT), a critical step in cancer metastasis (Zhang et al. 2020). Se‐POP‐21 has demonstrated an ability to induce apoptosis and inhibit metastasis in A549 cells by blocking EMT, suggesting potential as a low‐toxicity cancer treatment (Zhang, Zhang, Liu, et al. 2021). The study further investigated the anticancer mechanisms of Se‐POP‐3, specifically targeting gastric (MGC‐803) and colon cancer (HCT‐116) cells. The results showed that Se‐POP‐3 significantly reduced cell viability, induced apoptosis, inhibited cell migration and invasion, and disrupted the Bax/Bcl‐2 ratio, thus promoting apoptosis (Zhang, Zhang, Liu, et al. 2022). Additionally, Se‐POP‐3 inhibited EMT, reducing cancer cell insensitivity and metastatic potential. Mainly, Se‐POP‐3 exhibited selectivity, as it did not significantly affect the growth of normal cells (NCM460) within the tested concentration range.

Additionally, polysaccharide‐decorated Se^0^NPs derived from *Gracilaria lemaneiformis* (GLP) exhibited enhanced cellular uptake in U87 glioma cells compared to C6 cells. This selective uptake is attributed to the high expression of αvβ3 integrin on the U87 cell membranes, which strongly binds to GLP‐coated Se^0^NPs, suggesting targeted anticancer efficacy (Li, Shen, et al. 2019). Combining Se and polysaccharides is a promising approach to enhancing anticancer properties. *Paeonia lactiflora* ‐derived polysaccharide (PLP50‐1) formed nanoparticles with Se (PLP‐Se^0^NPs) that demonstrated more significant anti‐proliferative activity against A549 cells than PLP50‐1 alone (Wang et al. 2023). APS‐Se^0^NPs were synthesized from *Astragalus* polysaccharide, and in vitro anti‐hepatoma experiments showed that these composites significantly inhibited HepG2 cell proliferation in a dose‐dependent manner. They induced morphological changes, caused cell cycle arrest in the S phase, and ultimately triggered apoptosis in HepG2 cells through the mitochondrial pathway. Similarly, DP1‐Se^0^NPs induced apoptosis in HepG2 cells by causing DNA fragmentation, nuclear condensation, and cell cycle arrest at the S phase. The apoptotic pathways activated by DP1‐Se^0^NPs included the activation of caspase‐3, ‐8, and ‐9 activation, as well as FADD engagement.

**FIGURE 8:**
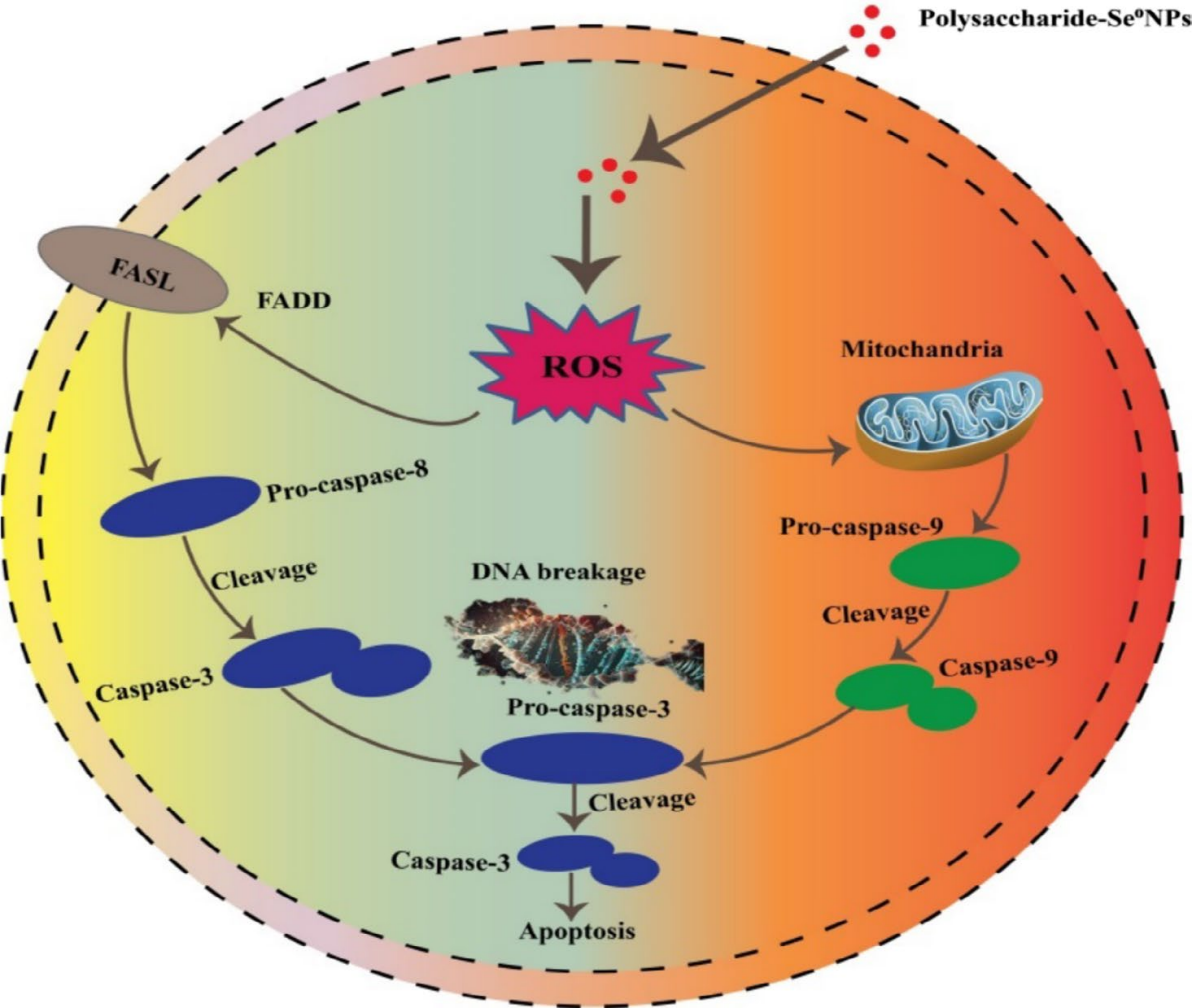
Polysaccharide Se^0^NPs from *Dictyophora indusiata* induce apoptosis in HepG2 cells by generating ROS. The ROS triggers both the extrinsic (FASL/FADD) pathway, leading to caspase‐8 activation, and the intrinsic (mitochondrial) pathway, leading to caspase‐9 activation. Caspase‐8 and caspase‐9 converge on caspase‐3, which initiates DNA fragmentation and ultimately results in cell apoptosis.

Additionally, DP1‐Se^0^NPs disrupted mitochondrial activity and increased ROS production, further accelerating apoptosis (Liao et al. 2015) (Figure 8). *Laminarin* polysaccharides decorated with Se nanoparticles (LP‐Se^0^NPs) exhibited significant cytotoxic effects against HepG2 liver cancer cells. Treatment with varying concentrations of LP‐Se^0^NPs led to an increase in the total apoptosis rate in cells. The cytotoxic effect is mediated through a mitochondria‐dependent apoptotic pathway, characterized by increased levels of the pro‐apoptotic proteins Bax and cleaved caspase‐9, and a reduction in the anti‐apoptotic protein Bcl‐2. This shift in apoptotic regulators suggests that LP‐Se^0^NPs induce cell death by disrupting mitochondrial integrity and activating intrinsic apoptotic signaling, thereby enhancing their effectiveness against HepG2 cells (Cui et al. 2019).

### 5.3 Immune Enhancement Activity

**FIGURE 9:**
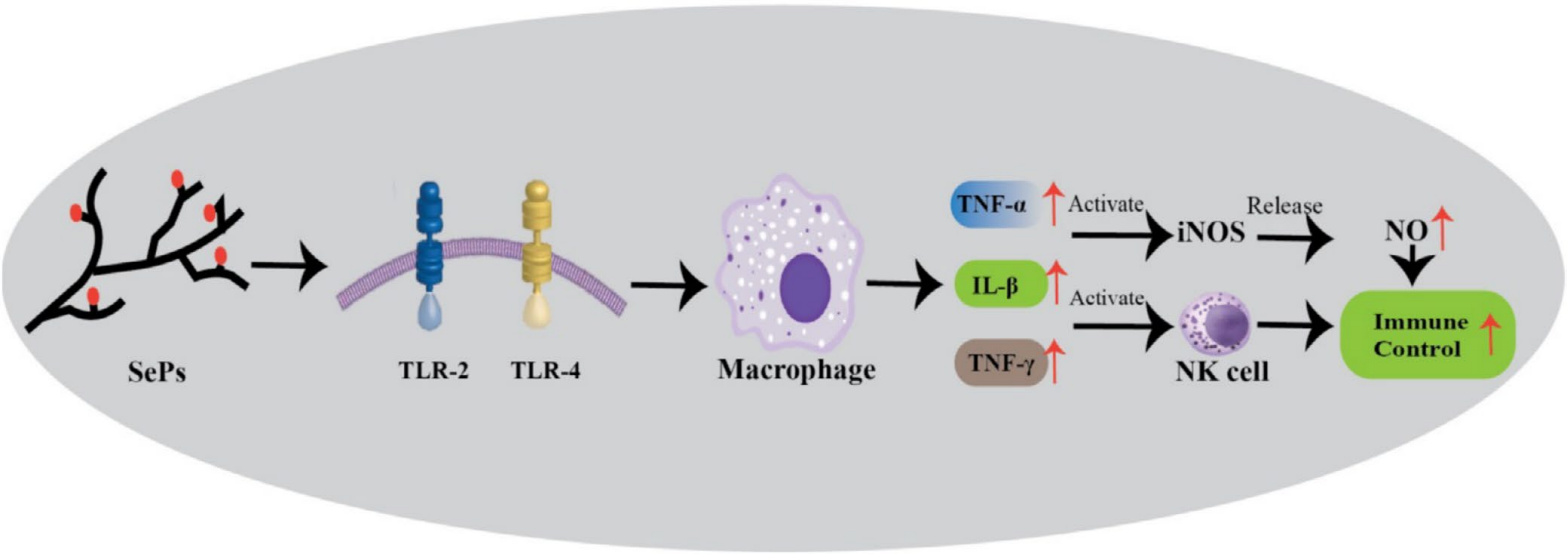
SePs interact with TLR‐2 and TLR‐4 receptors on macrophages, leading to the activation of macrophages and the release of cytokines (TNF‐α, IL‐β, TNF‐γ). These cytokines stimulate NK cells and increase the production of NO via iNOS, strengthening immune control.

Immune enhancement is a key benefit of SePs, strengthening the body's defense against infections, inflammatory diseases, and cancer. The growing interest in natural SePs has emphasized their potential for immune modulation. SePs have the potential to strengthen immune defenses through mechanisms such as cytokine modulation, antioxidant enzyme activity, and immune receptor engagement, emphasizing as natural agents for immune support and disease prevention (Figure 9). Modified *Angelica sinensis* polysaccharides (ASP‐Se^0^NPs) were shown in vivo experiments to effectively prevent CCl_4_‐induced acute liver injury by improving liver function, inhibiting oxidative stress and inflammatory responses, and thereby reducing liver pathological damage (Xu et al. 2024). In another study, SePs derived from *Platycodon grandiflorum* were found to enhance immune function through several mechanisms. In vitro, SePs increased cell viability, promoted natural killer (NK) cell activity, and enhanced cytotoxic T lymphocyte (CTL) activity, while also elevating the production of inflammatory cytokines (TNF‐α, IFN‐γ, IL‐2, and IL‐12) and immunoglobulins (IgA and IgG). In vivo, SePs facilitated the recovery from cyclophosphamide (CP)‐induced immunosuppression by restoring the counts of white blood cells, neutrophils, and lymphocytes, as well as increasing the production of inflammatory cytokines and immunoglobulins. Moreover, SePs reduced CP‐induced damage in the spleen and thymus, suggesting their potential as an immune‐enhancing agent (Noh et al. 2019).

Similarly, SeEPS obtained from *Enterobacter cloacae* promoted weight gain and improved feed efficiency in mice without causing liver toxicity. SeEPS enhanced antioxidant capacity and reduced MDA levels by upregulating genes involved in SeCys synthesis. This results in elevated selenoprotein levels, which play a key role in cellular defense and immune regulation (Cao et al. 2022). Selenylation modification of polysaccharides has been shown to enhance the immune‐enhancing activity of natural polysaccharides significantly. Se‐GFP‐22, derived from *G. frondosa*, demonstrated significant immunomodulatory effects through the TLR4‐mediated MAPK signaling pathway. Although Se‐GFP‐22 was non‐cytotoxic, it enhanced macrophage immune responses by promoting phagocytosis and upregulating key immune factors, including IL‐2, TNF‐α, IFN‐γ, and NO, along with their corresponding mRNA expressions. The increase in SOD activity suggested a protective mechanism against oxidative stress in macrophages. Significantly, blocking the TLR4 pathway suppressed macrophage activation, indicating that Se‐GFP‐22 activates macrophages specifically through the TLR4‐mediated MAPK pathway. This finding was further corroborated by western blot analysis and the use of MAPK‐specific inhibitors (Li et al. 2023). SePs from *Radix isatidis* (RIWP) increased SOD activity and GSH levels, which protected cells from oxidative damage and reduced the production of pro‐inflammatory molecules, including ROS, PGE₂, TNF‐α, IL‐6, and NO. This mechanism emphasizes RIWP's potential to manage lung inflammation by stabilizing oxidative and inflammatory pathways (Tao et al. 2021). For inflammatory bowel disease (IBD), selenized polysaccharides from *Ulva pertusa* (ulvan‐Se) demonstrated therapeutic potential in a mouse model of DSS‐induced IBD. Ulvan‐Se increased the expression of tight junction proteins, such as zonula occludens protein 1, occludin, and claudin‐1, thereby reinforcing the intestinal epithelial barrier. By preventing white blood cell infiltration and maintaining structural integrity, ulvan‐Se appears to provide both structural and immunological support, thereby mitigating IBD symptoms (Wang et al. 2021). Structural modifications due to Se integration influenced inflammatory responses in HK‐2 cells. Se‐DCSP significantly reduced inflammatory markers such as MCP‐1 (by approximately 1.7 times), NLRP3, and NO levels (by around 1.5 times) compared to DCSP (Zheng et al. 2024). The immunomodulatory effects of Se‐modified *S. sagittifolia* L. polysaccharide (Se‐PSSP) are primarily driven by its enhanced bioavailability and antioxidant properties, which are significantly improved through Se supplementation. This modification enhances the polysaccharide's solubility, facilitating improved interaction with immune cells and promoting key immune functions, including macrophage phagocytosis and the activation of T‐cells and NK cells. Moreover, Se‐PSSP helps regulate oxidative stress by modulating cytokine production, contributing to its antineoplastic and cytotoxic effects, which may enhance the immune system's ability to recognize and eliminate tumor cells (Feng et al. 2022). Se‐containing tea polysaccharide (ASeTP) alleviates ulcerative colitis (UC) by enhancing the colonic mucosal barrier through up‐regulation of tight junction proteins (occludin, claudin‐1, ZO‐1). It reduces pro‐inflammatory cytokines, increases antioxidant capacity in colon tissue, and raises Se content in the colon (Zhao et al. 2022).

### 5.4 Hypoglycemic Activity

**FIGURE 10:**
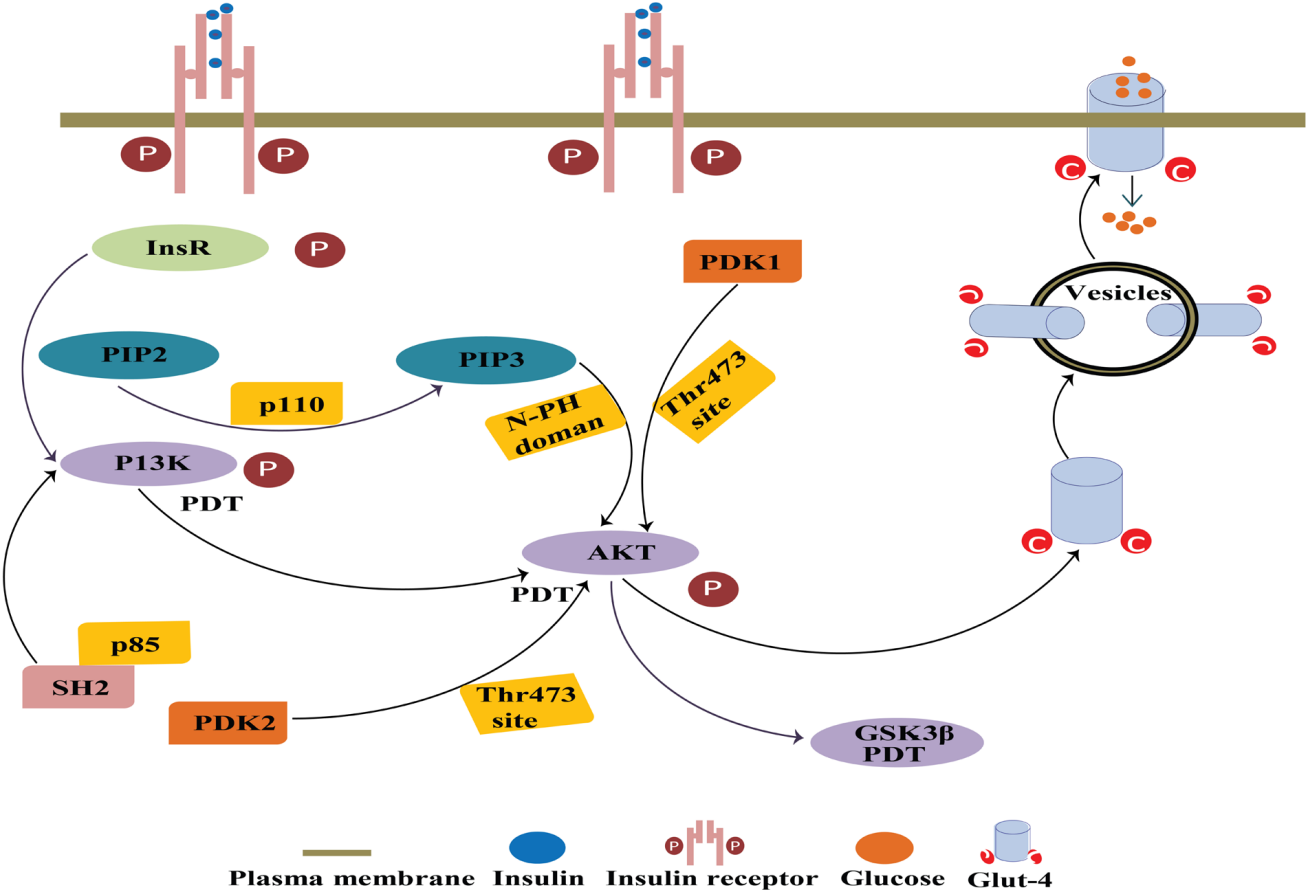
The potential role of SePs in modulating the insulin signaling pathway, identifying key drug targets such as insulin receptor substrates (InsR), phosphatidylinositol‐4,5‐bisphosphate (PIP2), and phosphatidylinositol 3,4,5‐trisphosphate (PIP3). The path involves InsR activation, which triggers downstream signaling through PI3K and Akt, ultimately promoting the translocation of Glut‐4 vesicles to the membrane for Glucose uptake. SePs may enhance this pathway, potentially improving insulin sensitivity and Glc metabolism.

Diabetes is a metabolic disorder marked by chronic hyperglycemia and insulin deficiency, leading to dysfunction across various tissues. A significant drawback of conventional hypoglycemic medications is the risk of hypoglycemia. However, SePs exhibit a unique regulatory effect on blood Glc, ceasing Glc reduction once normal levels are restored. Studies have shown that SePs from *Cyclocarya paliurus* and *Catathelasma ventricosum* effectively inhibit α‐glucosidase enzyme, which delays Glc synthesis and absorption, thereby helping to lower postprandial blood Glc in diabetic patients. This inhibition aligns with type 2 diabetes treatments targeting α‐glucosidase; however, SePs demonstrate a naturally dose‐dependent and regulated inhibition as observed in Se‐enriched *C. paliurus* (Liu, You, et al. 2017; Wang et al. 2022). A selenized derivative of polysaccharides from *S. pallidum* (Se‐SPP) demonstrated significantly more potent, noncompetitive inhibition of α‐glucosidase activity compared to unmodified SPP and the conventional hypoglycemic drug acarbose. This improved inhibition suggests that Se enrichment enhances the polysaccharide's ability to delay carbohydrate breakdown and Glc absorption, introducing a possible alternative with reduced side effects (Wang et al. 2019). *Spirulina* polysaccharide‐3 (SPS‐3) was selected for selenylation due to its high rhamnose and Glc content, which provided optimal Se binding efficiency. The resulting SeCSPS demonstrated significantly enhanced antihyperglycemic activity compared to native SPS‐3, primarily due to the structural alterations induced by Se binding (Qian et al. 2024). SePs also inhibit other key enzymes in Glc metabolism, such as α‐amylase, and activate the insulin signaling pathway, particularly the IRS‐PI3K‐Akt pathway, which is essential for cellular Glc uptake and insulin sensitivity (Duan et al. 2022), as shown in (Figure 10). Selenylation further enhances these effects, as shown in selenylated polysaccharides from *Momordica charantia* L. (Se‐MCPIIa‐1). This derivative demonstrated optimal hypoglycemic activity at a lower dosage (20 mg/kg body weight) than its unmodified form, showing that Se incorporation creates a synergistic hypoglycemic effect by combining the biological activity of both Se and polysaccharides (Ru et al. 2020).

GLPs‐stabilized Se nanoparticles (GLPs‐Se^0^NPs) demonstrated significant antidiabetic potential by strongly inhibiting α‐amylase and α‐glucosidase activity, key enzymes in starch and sugar metabolism. This inhibition delays polysaccharide breakdown and glucose absorption, highlighting their potential for managing postprandial blood glucose levels (Cao, Zhang, et al. 2021). Moreover, polysaccharides derived from *L. caerulea* L. pomace (LPP) exhibit significant hypoglycemic activity through dose‐dependent inhibition of both α‐amylase and α‐glucosidase, with effects comparable to acarbose. However, unlike acarbose, which is associated with adverse effects, LPP provides a natural alternative with fewer side effects, highlighting its potential for diabetes treatment (Fu et al. 2020). SePs are therefore suggested to hold potential as hypoglycemic agents by targeting multiple mechanisms, inhibiting glucosidase and amylase activity, modulating the insulin signaling pathway, and reducing oxidative stress, making them valuable for diabetes treatment with minimized side effects.

### 5.5 Heavy Metals Removal

**FIGURE 11:**
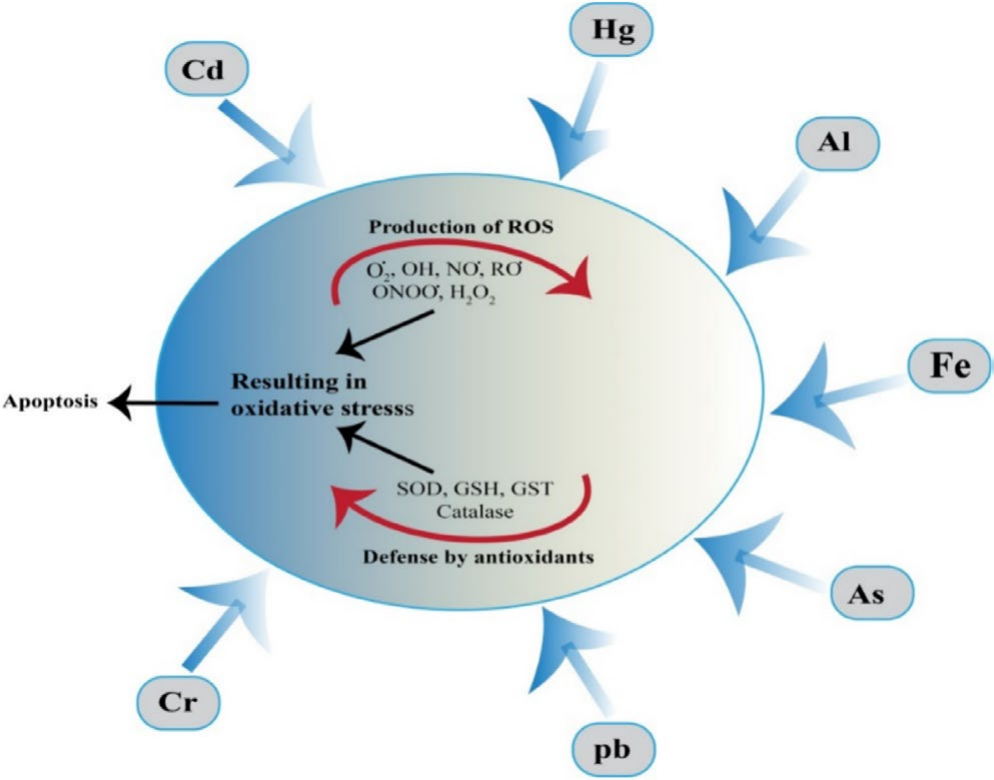
Shows how heavy metals, for example, Cd, Hg, Al, Fe, As, Cr, and Pb, induce reactive ROS in cells, leading to oxidative stress. Antioxidants such as SOD, GSH, GST, and Cat work to neutralize ROS. However, excessive ROS overwhelms these defenses, resulting in cell apoptosis. Figure also shows the balance between ROS production and antioxidant defense in response to heavy metal exposure.

Heavy metal toxicity poses serious health risks as toxic metals like mercury (Hg), cadmium (Cd), lead (Pb), and arsenic (As) disrupt normal metabolic processes in the body despite having no essential biological function. Under certain conditions, metals act as pseudo‐elements in the body and may disrupt metabolic processes. The interaction between heavy metals and cells, along with the balance between ROS production and the defensive role of antioxidants, is shown in (Figure 11). SePs have been shown to interact with toxic metals, helping in their removal and mitigating their harmful effects. Se‐enriched *Auricularia auricula* was evaluated for mercury detoxification in mice. Results showed that *A. auricula* significantly raised Se levels in organs such as the brain, heart, lungs, liver, kidneys, and blood while lowering Hg concentrations more effectively than Na_2_SeO_3_. This dual effect likely results from Se's ability to form stable Se‐Hg complexes, rendering them inactive and thus reducing mercury bioavailability and toxicity in the body (Hu et al. 2019).

Se‐SPP from *Spirulina platensis* has been synthesized and examined for its protective effects against Cd toxicity. Both in vitro and in vivo studies demonstrate that Se‐SPP provides substantially better protection against Cd toxicity than unmodified polysaccharides or Na_2_SeO_3_ alone, indicating that the covalent bonding of Se to the polysaccharide enhances its biological activity. This bonding may enhance the structural stability of Se‐SPP, allowing it to reduce oxidative damage effectively and bind toxic metals for safer excretion from the body (Zhou, Long, Wang, Zhu, et al. 2020). Similarly, Se‐SPP was investigated for its role in mitigating Cd‐induced toxicity by modulating gut microbiota and liver inflammation. The results suggest that Se‐SPP can mitigate hepatic inflammation by selectively targeting microbial populations associated with Cd‐induced inflammation, indicating a gut‐liver axis mechanism in managing toxicity (Zhou et al. 2022). Cd toxicity primarily affects the kidneys through oxidative damage, while SePs have demonstrated protective effects by activating antioxidant defense mechanisms. In renal tissue, bacterial extracellular SePs increased SOD and GSH‐Px activities while lowering MDA levels, thus preserving kidney function (Momeni et al. 2019). SePs' ability to enhance antioxidant enzymes likely mitigates the oxidative damage induced by Cd, as SOD and GSH‐Px play key roles in neutralizing ROS generated by Cd exposure. Dietary supplementation with SePs has shown similar benefits, maintaining physiological functions in Cd‐exposed rats by upregulating antioxidant defenses in the kidneys (Yang et al. 2021). SePs treatment has also shown benefits in mitigating Pb toxicity. In mice exposed to Pb, administration of agaric‐derived SePs demonstrated protective effects on the liver, likely by reducing ROS levels and enhancing antioxidant enzyme activity, thus protecting liver tissues from oxidative damage. As toxicity induces lipid peroxidation and inhibits antioxidant molecules and enzymes, Se plays a crucial role in activating GSH‐Px. Increasing both Se levels and GSH‐Px activity, SePs reduce the effects of As‐induced oxidative stress, supporting cellular integrity and reducing toxicity (Li, Yan, et al. 2019). SePs demonstrate promising protective effects against heavy metal toxicity by forming inactive complexes with metals, enhancing antioxidant defenses, and mitigating ROS production. They highlight their potential as therapeutic agents for heavy metal detoxification and antioxidant support.

### 5.6 Other Activities

In addition to the biological activities discussed, SePs exhibit a wide range of effects across multiple health domains. First, SePs demonstrate crucial antibacterial and antiviral activity. The rising prevalence of bacterial infections and antibiotic resistance has underscored the urgent need for new antibacterial agents. For example, *Enteromorpha prolifera* (Se‐LEP) has shown potent antibacterial action against plant pathogenic fungi and *Escherichia coli* . These enhanced antibacterial effects are attributed to Se's ability to disrupt microbial cell membranes, inducing oxidative stress in the pathogenic cells (Lv et al. 2018). Similarly, Se exopolysaccharides from *Fomes fomentarius*, selenylated with Na_2_SeO_3_, showed increased effectiveness against *Staphylococcus aureus* . The integration of Se appears to improve polysaccharide oxidative and membrane‐disruptive actions against bacteria (Alvandi et al. 2021). In antiviral applications, selenide polysaccharides were evaluated against the CVB3 enterovirus. SePs demonstrated greater efficacy and safety than ribavirin, a common antiviral, in inhibiting CVB3 at concentrations ranging from 0.015 to 0.500 g/mL. The enhanced antiviral effect of SePs may be attributed to their ability to modulate host cell oxidative responses, thereby reducing viral replication by limiting the available resources for viral growth (Yang et al. 2021).

Second, SePs contribute significantly to blood lipid regulation, addressing hyperlipidemia, which raises the risk of chronic heart disease. Clinical trials on hyperlipidemic patients have shown that konjac‐derived SePs capsules effectively reduce lipid levels and blood glucose, potentially influencing lipid metabolism pathways and enhancing insulin sensitivity (Song et al. 2022). Furthermore, the polysaccharides derived from *F. fomentarius* (PS, PS‐Se, SLN‐PS, SLN‐PS‐Se) have shown improvements in lipid profiles, suggesting their potential for managing hyperlipidemia by enhancing lipid clearance and supporting liver function (Keshavarz‐Rezaei et al. 2022). SePs have also shown cardioprotective impact, as evidenced by Se‐AVP derived from *Aloe vera* , which protects against myocardial ischemia–reperfusion (I/R) injury in rats. Using an in vivo I/R model, Se‐AVP was found to elevate natural antioxidant levels, thereby protecting cardiac tissue from oxidative stress and preventing ischemia‐induced cardiac damage. The mechanism likely involves Se's capacity to increase intrinsic antioxidant defenses, such as SOD and GSH‐Px, which mitigate ROS levels during cardiac stress (Yang et al. 2017).

Third, currently, an increasing number of studies are dedicated to understanding the mechanisms of aging and exploring potential strategies to delay its progression. Aging, attributed to oxidative stress and free radical accumulation, may be slowed by SePs' anti‐aging properties. The anti‐aging properties of SePs are associated with various mechanisms, including enhanced antioxidant capacity, regulation of age‐related gene expression, and strengthened immune function. An analysis of epidemiological studies revealed that insufficient dietary intake of Se and Zn can lead to cognitive impairments in aged individuals (Steinbrenner and Klotz 2020). Se supplementation reverses age‐related cognitive decline (Leiter et al. 2022). The beneficial skincare and anti‐aging effects were observed following the external application of Se‐enriched fermented mung beans (Wei et al. 2022).

Fourth, Se‐APS from *M. sativa* L. stems has demonstrated enhanced neuroprotective effects compared to its unmodified polysaccharide, highlighting its potential in neurological applications. The neuroprotective benefits may arise from Se's role in protecting neurons against oxidative damage and supporting neuronal function, placing Se‐APS as a promising candidate for drug development and neurodegenerative research (Liu et al. 2020). The neuroprotective effect of seleno‐polymannuronate (Se‐PM) was studied, and it was found that Se‐PM significantly inhibits Aβ_1‐42_ oligomer aggregation and reduces the expression of neurodegeneration‐related proteins (APP and BACE1) in N2a‐sw cells. Additionally, Se‐PM lowered cytochrome c levels, decreased the Bax/Bcl‐2 ratio, and enhanced mitochondrial membrane potential, collectively preventing cell apoptosis and promoting cell survival (Bi et al. 2020).

## 6 Structure–Activity Relationship of SePs

Currently, we may not be able to elucidate the structure–activity relationship of SePs entirely; however, by referencing current research, we aim to provide insights into this relationship. The diverse biological activities of SePs have been extensively documented in the scientific literature. However, due to the complexity of their molecular structures characterized by high molecular weights, complex conformations, particle size, Se content, and various forms of Se incorporation, understanding the precise structure–activity relationship remains challenging. The following aspects discuss the structure–activity relationship of SePs. (1) Se incorporation: Se incorporation into polysaccharides can significantly influence their structural and biological activity. For instance, two Se‐enriched tea polysaccharides (Se‐TPS), CSe‐tps1 and ASe‐tps1, demonstrate how different Se incorporation methods can impact their structure and activity. The polysaccharides were found to have distinct structural characteristics, with Se in CSe‐tps1 replacing the OH group at C‐6 as a selenyl ester, while in ASe‐tps1, Se was incorporated at both C‐1 and C‐6 positions, forming Se‐H bonds. Structural analyses revealed that both Se‐TPSs exhibited a triple helix structure but differed in heat release properties, crystal morphology, and α‐glucosidase inhibition activity. These differences in structure led to varied hypoglycemic activities, demonstrating that Se incorporation and the resulting polysaccharide structure significantly influence their biological activity (Zhu, Yu, et al. 2020). Similarly, the three purified SePs components from *Cordyceps militaris* exhibited a triple‐helix structure, with those having more branches demonstrating higher biological activity (Liu, Zhu, et al. 2017). In the structure–activity relationship of HP2‐Se^0^NPs (hawthorn polysaccharide Se nanoparticles), incorporating Se into the hawthorn polysaccharide structure is key to enhancing its antioxidant activity. The presence of Se increases the electron density on the polysaccharide, improving its ability to scavenge free radicals, such as DPPH, ˙OH, and ABTS, and enhancing its antioxidant properties compared to unmodified HP2 (Sun et al. 2023). (2) Effect of branching and particle size: Three purified SePs components from *C. militaris* exhibited a triple‐helix structure, with those having more branches demonstrating higher biological activity (Liu, Zhu, et al. 2017). Se‐POP‐3, a linear β‐D‐glucan with specific branching patterns, demonstrates how the arrangement and type of glycosidic linkages can influence the overall properties and biological interactions of SePs (Zhang, Zhang, Liu, et al. 2022). Structural modifications, such as changes in surface morphology and particle size, also play a crucial role in determining their behavior. SPS‐Se^0^NPs, with sizes ranging from 54.35 to 123.04 nm, exhibit unique properties influenced by their nanoparticle size. SPS‐Se^0^NPs interact differently with biological systems, thereby modulating their activity (Hu et al. 2020). Another example is *Laminaria* polysaccharide‐stabilized Se nanoparticles (LP‐Se^0^NPs), which exhibit non‐covalent interactions between the LP and Se^0^NPs, thereby enhancing the stability and functional properties of the nanoparticles. A smaller particle size (81.41 nm) and higher Se content (653.91 mg/g) increase the surface area and reactivity of Se^0^NPs, resulting in improved antioxidant activity. These characteristics, along with increased stability and reduced toxicity, make LP‐Se^0^NPs effective as antioxidant and hypoglycemic agents (Yang et al. 2024). The antioxidant properties of Tribonema polysaccharide (TP)‐Se^0^NPs are closely linked to their structural characteristics. The increased Se content and smaller particle size significantly contribute to their enhanced antioxidant activity. Furthermore, non‐covalent interactions between TP and Se^0^NPs improve their stability and dispersibility, which in turn enhances their antioxidant effects and suggests a possible synergistic mechanism (Yang et al. 2025). The morphology of Se^0^NPs, such as those stabilized by *Astragalus* polysaccharides, is another example that enhances their stability and functional properties, influencing their cellular activities (Jiao et al. 2022). (3) Molecular weight and monosaccharide composition: The molecular weight of SePs plays a significant role in their biological activity. Se‐RLFP‐II, with a lower molecular weight (1.6 × 10^3^ Da) than Se‐RLFP‐I (2.4 × 10^3^ Da), exhibited enhanced biological activity, including better antioxidant and neuroprotective effects. The lower molecular weight Se‐RLFP‐II demonstrated superior radical scavenging activity and more effective protection of SH‐SY5Y cells from H_2_O_2_‐induced damage. This indicates that decreasing the molecular weight of SePs may improve their biological efficacy, possibly by enhancing their absorption and interaction with biological systems (Liu et al. 2022). To enhance the antioxidant activity of SePs, selenylated polysaccharides from *E. prolifera* were degraded to a lower molecular weight using a free‐radical degradation method involving H_2_O_2_ and ascorbic acid. Structural modifications are likely linked to the improved physiological activities of SePs (Lv et al. 2018). SeTPS‐1 and SeTPS‐2 reveal that SeTPS‐1, with a simple sugar composition (Glc and Gal) and a higher molecular weight (1.7 × 10^4^ Da), exhibited more potent antioxidant activity and better DNA damage protection than SeTPS‐2, which has a more complex sugar composition and a smaller molecular weight (1.3 × 10^4^ Da). The higher molecular weight of SeTPS‐1 contributes to its enhanced stability and more effective cellular interactions. In contrast, the complex sugar structure and smaller molecular weight of SeTPS‐2 likely hinder its biological activity (Gu et al. 2020). Se‐enriched polysaccharides such as Se‐PPS1 and Se‐PPS3 demonstrate that their sugar composition and glycosidic linkages significantly influence their structural properties and efficiency in biological processes, further emphasizing the importance of sugar composition and linkage type in modulating biological activity (Xiang et al. 2022). Similarly, the specific sugar composition and glycosidic bonds of SeCPS‐II influence its ability to induce apoptosis in cancer cells (e.g., SKOV‐3 cells). The apoptosis is mediated through the p53‐Bax‐caspase signaling pathway. The structure of the SePs may affect their ability to interact with cellular pathways, leading to tumor cell death (Sun et al. 2018). (4) Se content and forms: Se content is generally considered an important factor in the biological activity of SePs; increasing Se content does not necessarily lead to directly enhancing their biological activity. Although some studies show that higher Se content is associated with better biological activity, this is not always the case. For example, increasing the dose of SePs increases both the Se and polysaccharide portions, allowing both components to influence the biological activity. Different doses of SeO_3_^2−^ were added to produce two selenized polysaccharides from *Ulmus pumila* L., both of which had the same core polysaccharide structure but varied in Se content. The anti‐inflammatory activities of the polysaccharides were evaluated, and both significantly inhibited NO production in LPS‐induced RAW 264.7 cells. The polysaccharide with a higher Se content showed a stronger effect (Lee et al. 2018). Similarly, the structure–activity relationship shows that incorporating Se into the polysaccharide structure, specifically at the C‐6 position as SeO_3_^2−^, is crucial for enhancing the antiproliferative properties of SeASP. The higher the Se content (∼22,400 μg/g), the stronger the antitumor activity, as evidenced by the lower IC_50_ value (24.35 μg/mL) against HepG2 cells. Additionally, the increased Se content induces structural changes in the polysaccharide, leading to spherical and rod‐shaped conformations, which are linked to improved biological function (Zhu, Hu, et al. 2020).

Similarly, the SeASPs show that Se content directly correlates with their anti‐tumor activity. Higher Se levels (13,030 μg g^−1^) in SeASP enhance apoptosis and cell cycle arrest in tumor cells while maintaining low cytotoxicity to normal cells. This effect is linked to increased mitochondrial disruption, caspase activation, and immune modulation (Liu et al. 2021). SeEPS demonstrate how Se content can enhance enzyme activities, modify the structural behavior of polysaccharides, and increase their overall stability and bioavailability (Cao et al. 2022). The various Se forms (Se^0^, SeO_3_^2−^, SeO_4_^2−^, SeMet, and Se‐methyl‐SeC) in polysaccharides are essential in optimizing their structural and functional properties. Modifications, such as changes in molecular weight or monosaccharide composition, enhance the capabilities of polysaccharides, improving their applications in various therapeutic and environmental contexts (Wang, Ji, et al. 2024).

## 7 Emerging Therapeutic and Industrial Applications of SePs

SePs have gained significant interest as functional supplements due to their high bioavailability, minimal side effects, and diverse health benefits (Chang and Liu 2024). Incorporating SePs into the diet enhances Se absorption and supports essential health functions, including immune support, cancer prevention, and the reduction of oxidative stress, providing a holistic approach to Se intake (Li et al. 2021). SePs derived from various sources can be utilized in nutraceutical supplements in the near future, as ongoing research continues to highlight their high bioavailability, antioxidant properties, and health benefits, including immune support, cancer prevention, and reduction of oxidative stress. For example, SeCPS‐II and Se‐POP‐1 have shown potential for inclusion in Se‐containing dietary products due to their enhanced Se bioavailability and antioxidant properties, which contribute to stress reduction and general health maintenance (Ma et al. 2018; Sun et al. 2018). Selenylation of the pulp polysaccharide from *Rose laevigata Michx* fruit (PPRLMF‐2) significantly enhances its antioxidative and immunoregulatory activities, making it a promising candidate for dietary and nutraceutical applications. The modified version, Se‐PPRLMF‐2, holds potential as a valuable source of dietary Se supplements, immune system enhancers, and antioxidants. This modification improves the functional and therapeutic properties of the polysaccharide (Zhan et al. 2022). Similarly, SePs derived from various natural sources are increasingly utilized in nutraceutical supplements. For example, Se‐LMW‐GFP extracted from *G. frondosa* seed bodies shows potential in anti‐gastric cancer applications, while selenized chestnut polysaccharides (CP‐Se) and Se‐enriched lotus root polysaccharides have emerged as candidates for nutraceutical products, supporting mainstream production of Se‐enriched items (Huo et al. 2024; Zhang, Wang, et al. 2022).

Furthermore, Se‐PCS from *Camellia oleifera* shows protective effects against Mg toxicity by modulating the insulin/IGF‐1 pathway, highlighting the therapeutic potential of SePs in toxicology (Chen et al. 2022). The unique biological properties of SePs, including their ability to bind environmental toxins, also make them ideal for nutraceuticals aimed at detoxification and stress management. SePs from *H. serotina* are recognized for their antioxidant and metal‐binding capacities, which offer protective benefits against environmental toxins, making them suitable for health supplements and detox products (Wang, Li, and Wang 2018).

Additionally, TP‐Se^0^NPs (Tribonema polysaccharide‐stabilized Se nanoparticles) have shown significant promise as functional additives or pharmaceutical supplements, with demonstrated antioxidant properties and synergistic effects, offering the potential for use in dietary supplements to combat oxidative stress. SeCSPS, derived from *S. platensis* polysaccharide‐3 (SPS‐3), demonstrates higher antihyperglycemic activity than SPS‐3, making it a valuable organic Se supplement for nutraceutical products (Qian et al. 2024). Furthermore, innovations in Se^0^NPs technology are opening new possibilities. For example, Cts‐Se^0^NPs show potential for various antibacterial applications in medicine and dentistry. This solution could disinfect medical devices, serve as a mouthwash for periodontal diseases, and act as an anti‐caries agent (Rangrazi et al. 2020). RMLP‐Se^0^NPs also show potential as antioxidant compounds, making them suitable for nutraceutical ingredients and feed supplements to enhance oxidative stability and promote health benefits (Jha et al. 2022). TP‐Se^0^NPs show significant promise as antioxidant compounds, making them ideal for use as functional additives or pharmaceutical supplements, where the β‐1,3‐glucan structure of *Tribonema* polysaccharide (TP), combined with higher Se content and reduced particle size of TP‐Se^0^NPs, enhances their antioxidant activity and overall biological efficacy (Yang et al. 2025). With advancements in biofortification and selenization, SePs are well‐positioned to play a crucial role in the development of nutraceuticals. Their incorporation into health products provides a natural means of supporting immune health, cellular protection, and detoxification. As research progresses, SePs are expected to become integral to developing dietary products with broad preventive and therapeutic applications, solidifying their value in the nutraceutical market.

## 8 Conclusions and Perspective

This review highlights the increasing potential of SePs as bioactive compounds with a diverse range of health benefits. Organic Se, particularly in the form of SePs, offers enhanced bioavailability compared to inorganic Se sources, making it a valuable addition to nutritional and therapeutic products. SePs not only improve Se absorption but also mitigate the risks associated with the narrow therapeutic window between beneficial and toxic doses. Their integration into dietary supplements is expected to increase as health awareness continues to grow, based on current research trends.

However, natural SePs often contain low levels of Se, highlighting the need for synthetic methods to produce high‐Se SePs that meet market demand. Selenylated polysaccharides achieve higher Se content, maintaining structural similarities to their natural counterparts while improving their biological efficacy. Key to optimizing SePs' biological activities is understanding their structure–activity relationship, as structural modifications, such as in corporSeation at specific sites or the formation of seleno‐groups, can enhance their therapeutic potential. Continued research into the biosynthesis, structural characterization, and emerging applications of SePs is crucial. Such efforts will help refine the production and utilization of SePs in nutraceuticals and pharmaceuticals, further advancing their role in promoting human health.

Future research should address key areas: (a) Comparative studies on the toxicity and efficacy of natural versus selenylated SePs are essential for safe therapeutic applications. (b) Advanced techniques, such as transcriptomics profiling, can be employed to elucidate the transformation of inorganic to organic Se, enhancing our understanding of SePs biosynthesis at the molecular level in plants. (c) Research should focus on improving the bioavailability, targeted delivery, and therapeutic efficacy of SePs, as well as exploring innovative carriers and their potential applications in disease‐specific functional foods. (d) The structure–activity relationship of SePs, comparing natural and selenylated forms, should be examined to identify structural modifications that enhance efficacy and safety. Additionally, understanding how these modifications impact bioavailability and targeted delivery will optimize SePs for therapeutic and functional food applications.

## Author Contributions

**Shahidin:** conceptualization (equal), data curation (equal), formal analysis (equal), methodology (equal), software (equal), validation (equal), visualization (equal), writing – original draft (equal), writing – review and editing (equal). **Yilong Wu:** conceptualization (equal), formal analysis (equal), methodology (equal), software (equal), supervision (equal), writing – original draft (equal), writing – review and editing (equal). **Yan Wang:** conceptualization (equal), data curation (equal), methodology (equal), software (equal), writing – original draft (equal), writing – review and editing (equal). **Pengyan Zhu:** conceptualization (equal), data curation (equal), software (equal), writing – original draft (equal), writing – review and editing (equal). **Taixia Chen:** conceptualization (equal), software (equal), visualization (equal), writing – original draft (equal), writing – review and editing (equal). **Xuanjun Wang:** conceptualization (equal), funding acquisition (equal), methodology (equal), project administration (equal), supervision (equal), writing – original draft (equal), writing – review and editing (equal). **Chengting Zi:** conceptualization (equal), data curation (equal), formal analysis (equal), funding acquisition (equal), investigation (equal), methodology (equal), supervision (equal), writing – original draft (equal), writing – review and editing (equal).

## Conflicts of Interest

The authors declare no conflicts of interest.

## Data Availability

Data sharing not applicable to this article as no datasets were generated or analysed during the current study.
